# Aminoquinolones and Their Benzoquinone Dimer Hybrids as Modulators of Prion Protein Conversion

**DOI:** 10.3390/molecules27227935

**Published:** 2022-11-16

**Authors:** Amanda Rodrigues Pinto Costa, Marcelly Muxfeldt, Fernanda da Costa Santos Boechat, Maria Cecília Bastos Vieira de Souza, Jerson Lima Silva, Marcela Cristina de Moraes, Luciana Pereira Rangel, Tuane Cristine Ramos Gonçalves Vieira, Pedro Netto Batalha

**Affiliations:** 1 Instituto de Química, Universidade Federal Fluminense, Niterói 24020-141, RJ, Brazil; 2 Faculdade de Farmácia, Universidade Federal do Rio de Janeiro, Rio de Janeiro 21941-902, RJ, Brazil; 3 Instituto de Bioquímica Médica Leopoldo de Meis, Instituto Nacional de Ciência e Tecnologia de Biologia Estrutural e Bioimagem, Universidade Federal do Rio de Janeiro, Rio de Janeiro 21941-902, RJ, Brazil

**Keywords:** quinone, oxoquinoline, quinolone, scrapie, prion

## Abstract

Prion Diseases or Transmissible Spongiform Encephalopathies are neurodegenerative conditions associated with a long incubation period and progressive clinical evolution, leading to death. Their pathogenesis is characterized by conformational changes of the cellular prion protein—PrP^C^—in its infectious isoform—PrP^Sc^—which can form polymeric aggregates that precipitate in brain tissues. Currently, there are no effective treatments for these diseases. The 2,5-diamino-1,4-benzoquinone structure is associated with an anti-prion profile and, considering the biodynamic properties associated with 4-quinolones, in this work, 6-amino-4-quinolones derivatives and their respective benzoquinone dimeric hybrids were synthesized and had their bioactive profile evaluated through their ability to prevent prion conversion. Two hybrids, namely, 2,5-dichloro-3,6-bis((3-carboxy-1-pentyl-4-quinolone-6-yl)amino)-1,4-benzoquinone (**8e**) and 2,5-dichloro-3,6-bis((1-benzyl-3-carboxy-4-quinolone-6-yl)amino)-1,4-benzoquinone (**8f**), stood out for their prion conversion inhibition ability, affecting the fibrillation process in both the kinetics—with a shortening of the lag phase—and thermodynamics and their ability to inhibit the formation of protein aggregates without significant cytotoxicity at ten micromolar.

## 1. Introduction

Prions are proteins capable of undergoing conformational changes from their cellular isoform (PrP^C^) with a soluble and predominantly α-helical structure into their infectious isoform (PrP^Sc^) with an insoluble, β-sheet rich, and protease-resistant structure. PrP^C^ is encoded by a single gene known as PRNP and is expressed in different tissues, with a higher concentration in the central nervous system in the postsynaptic membranes of neurons [1]. 

There are two main models explaining the mechanisms of prion conformational change. One is the nucleation model, in which PrP^C^ converts, through a spontaneous conformational equilibrium, to the infecting PrP^Sc^. Although the conversion thermodynamics favors PrP^C^ conformation, the equilibrium can be shifted due to still unknown factors, leading to the formation of insoluble polymeric protein aggregates analogous to crystal nucleation. The second model is the refolding model, which proposes that once PrP^Sc^ is generated, it may serve as a template and induce further conformational changes in other PrP^C^ molecules, leading to protein aggregation. In both models, prion aggregates levels slowly increase, and they are deposited in neuronal tissues, causing the emergence of neurodegenerative symptoms and neuron death by apoptosis [2,3].

Prion Diseases, also known as Transmissible Spongiform Encephalopathies (TSEs), can affect diverse animals, including humans, and have no cure, being invariably fatal. In addition, they are infectious and can be transmitted, for example, through infected food ingestion or surgical instruments. Such diseases’ social and economic impacts make developing chemotherapeutic agents for treating TSEs urgent. Such drugs could, at first, act through different mechanisms, such as by modulating PrP^C^ expression and biosynthesis, stabilizing PrP^C^ through direct interaction with the protein, thus inhibiting the formation of PrP^Sc^, or decreasing the levels of prion aggregates directly [4,5,6,7,8,9].

Studies focusing on the development of new anti-prion agents or the identification of hits from virtual screening have grown in recent years, which can be justified by the development of more robust and sensitive methods to detect the modulatory effect of small molecules on prion conversion, using in silico, in vitro, and in vivo models [10,11].

**Figure 1:**
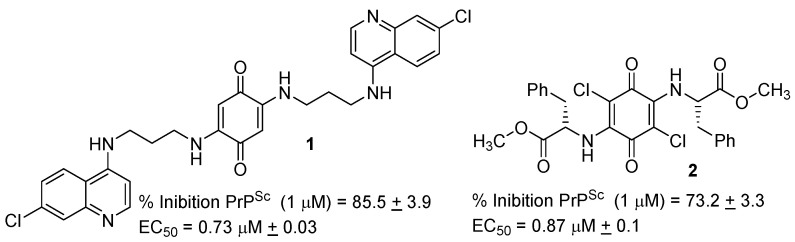
2,5-Diamino-1,4-benzoquinone derivatives, containing aminoquinoline (**1**) and phenylalanine (**2**) moieties, with anti-prion activity [12,13].

The 2,5-diamino-1,4-benzoquinone structure is a promising scaffold for the design of new substances with anti-prion profiles. In this context, two examples can be highlighted. Derivatives **1** and **2**, shown in Figure 1, containing a 4-aminoquinoline [12] and an α-aminoester [13] moiety, respectively, have presented excellent anti-PrP^Sc^ activity in vitro.

On the other hand, nitrogen-containing heterocycles are present in various bioactive structures. Such substances are associated with interesting physicochemical properties, including a high molecular dipole moment, susceptibility to ionization in a physiological medium, and the ability to perform polar and hydrogen bond interactions. Such characteristics make them structures of great importance in the Medicinal-Chemistry field and are continuously studied by the scientific community [14,15]. The development of new versatile synthetic methods for the construction of these molecular systems [16,17,18,19], the discovery of new substances with broad bioactive profiles [18,20], and the evaluation of their interaction mechanisms with different pharmacological receptors [20] are examples of research that has been developed over the last decades in this context.

**Figure 2:**
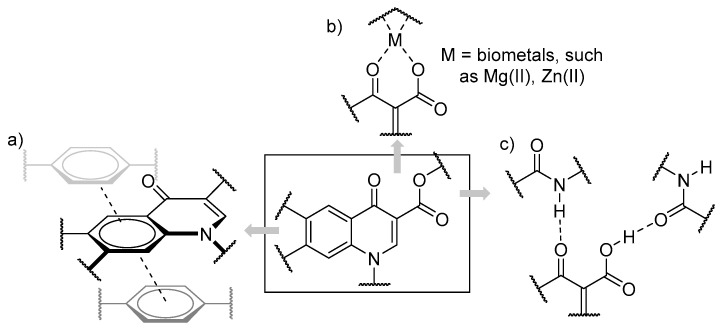
4-Quinolone motif and possible intermolecular interactions with biomolecules. (**a**) π-π Stacking interactions. (**b**) Chelating property with biometals. (**c**) Hydrogen bonds.

Despite their most common clinical application as antibacterials [21], 4-quinolone derivatives have also been described in numerous studies that demonstrate non-classical bioactivities, including anticancer [22,23], antiviral [24,25], antiparasitic [26,27], and antifungal [28]. These are biologically versatile substances due to their unique and easily functionalized structure. They may interact with different biological targets through intermolecular interactions, including hydrogen bonds, biometal chelation, and π-π stacking interactions (Figure 2) [29]. The biodynamic properties of these substances prompt us to consider their application as anti-prion agents.

Thus, contributing to the search for substances with chemotherapeutic potential against TSEs, we report the synthesis and anti-prion activity of 6-amino-4-quinolone derivatives and their 1,4-benzoquinone dimer hybrids and demonstrate through RT-QuIC, using in vitro-produced fibrils, their ability to inhibit prion aggregation.

## 2. Results and Discussion

### 2.1. Synthesis

**Scheme 1:**
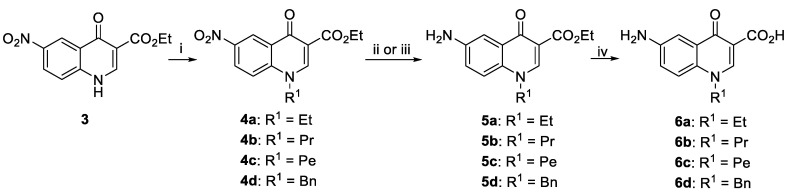
The synthetic route toward aminoquinolones **5a**–**d** and **6a**–**d**. i: (1) K_2_CO_3_, DMF, r.t. (2) alkyl halide (bromoethane, 1-bromopropane, 1-bromopentane or benzyl chloride), DMF, 80 °C, 24 h; ii: H_2_, Pd/C, EtOH; iii: Fe, NH_4_Cl aq., reflux; iv: (1) NaOH, EtOH, r.t., 24 h. (2) HCl aq. pH = 5–6.

Compounds **3**, **4a–d** [27], **5a–d,** and **6a** [30] were synthesized as described in previous works. Additionally, 6-amino-4-quinolone-3-carboxylic acids **6b**–**d** were prepared in the same way as described for **6a**, as shown in Scheme 1.

**Scheme 2:**
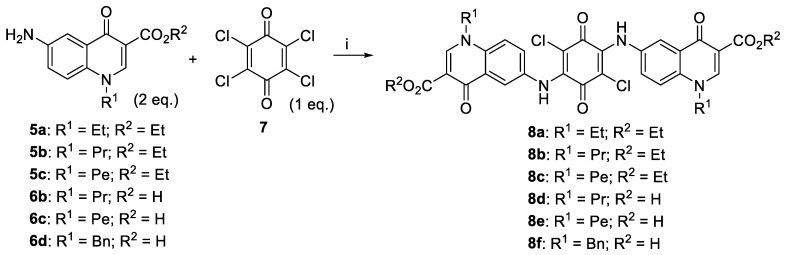
Synthetic strategy for the preparation of dimers **8a**–**f.** i: EtOH, 60 °C, 24 h.

6-Amino-4-quinolones **5a**–**c** and **6b**–**d** were successfully applied to the dimerization reaction via condensation with chloranil (**7**) in a stoichiometric ratio of 2:1, respectively (Scheme 2), following the protocol already described for the preparation of different 2,5-diamino-1,4-benzoquinone systems [12,13].

Substances **4a**–**d, 5a**–**d,** and **6a** had their structures properly characterized in previously published reports [27,30]. Substances **6b**–**d** and **8a**–**f** had their structures confirmed by analyzing their IR, ^1^H NMR, and ^13^C-APT NMR spectra. The analysis of their HRMS spectra also confirmed their molecular formulae. These data are described in the experimental section. The spectral data of these compounds are available in the Supplementary Materials File.

### 2.2. Biological Assays

PrP aggregation is associated with the pathology of prion diseases in which we can find fibrillar or amorphous aggregates. PrP^Sc^ can induce PrP^C^ structure conversion leading to the formation of auto-propagative aggregates [3]. This characteristic is involved in the infectious nature of prion diseases, but it is also observed in other conditions related to protein misfolding [31,32]. In vitro approaches can be used to investigate this auto-propagative ability, and the RT-QuIC assay is one of the most reliable for TSE diagnosis and therapeutic molecule screening [11,33].

Here, we performed the RT-QuIC assay to investigate the inhibitory effect of the synthesized compounds. In vitro fibrils produced under denaturing conditions induced PrP23-231 conversion and seeded its aggregation [26,34]. Previously, J8, a trimethoxychalcone, exhibited an inhibitory effect of prion conversion through this assay and inhibited approximately 50% of recombinant PrP conversion at 1 μM [33]. Table 1 shows the residual percentages of PrP conversion after treatment with compounds **5a–d**, **6a–d**, and **8a–f**.

**Figure 3:**
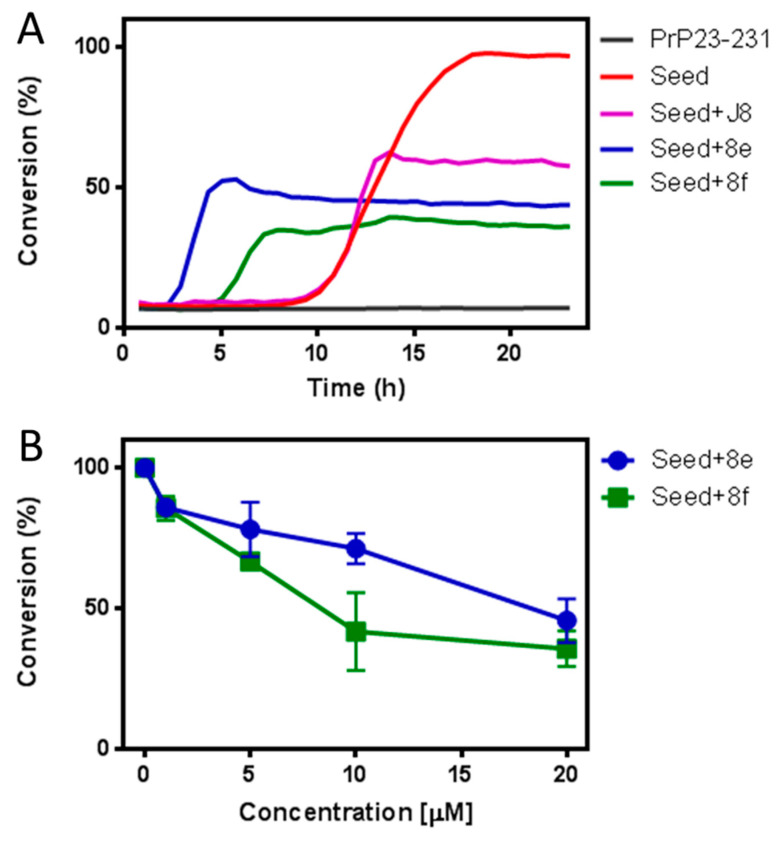
**8e** and **8f** decrease PrP seeding conversion and aggregation into fibrils. (**A**) RT-QuIC reactions with recombinant PrP23-231 (4 μM) as substrate in the absence (black line, negative control) or presence of PrP23-231 fibril seed (4%) (red line, positive control). The conversion was normalized to the values for the positive control (100%) and the negative control (0%). All compounds were incubated in the starting reaction at 20 μM. All conditions were performed in quadruplicate. Representative data of three independent experiments. (**B**) Conversion after RT-QuIC reactions of recombinant PrP23-231 with different compound concentrations. Data are represented as the mean of three independent experiments, and error bars represent the standard deviation.

**Figure 4:**
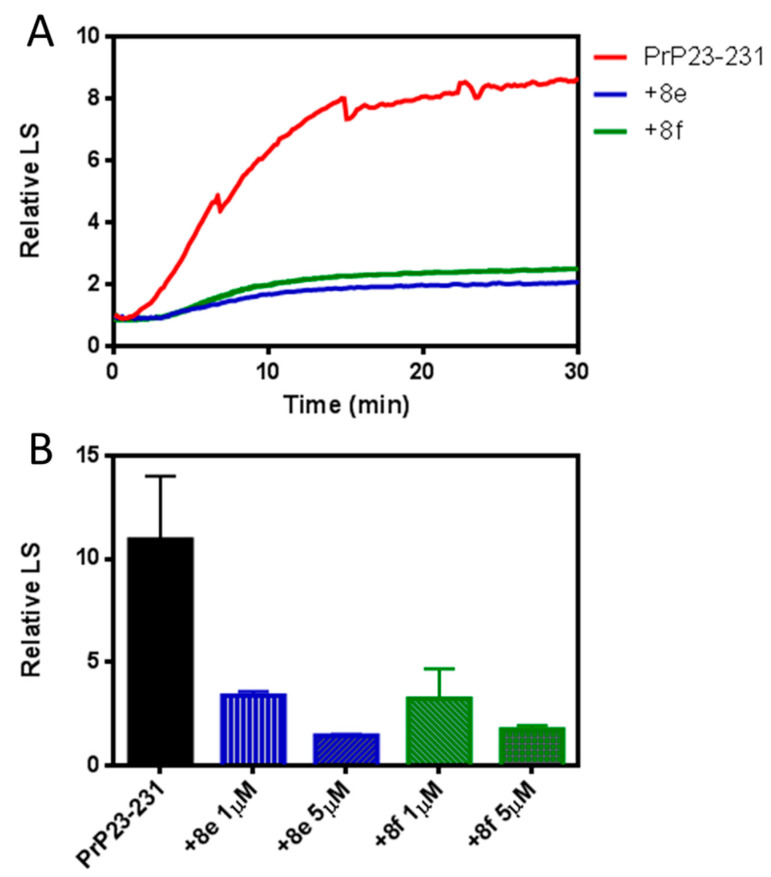
**8e** and **8f** decrease PrP aggregation induced by high temperature. (**A**) Recombinant PrP23-231 (1 μM) was incubated at 60 °C in the absence (red line) or the presence of compounds (1 μM), and light scattering (LS) was monitored over time. LS was represented relative to initial LS values at 25 °C. Representative data of three independent experiments. (**B**) Comparison of PrP23-231 steady-state aggregation at 60 °C (red bar) and in the presence of compounds at 1:1 (1 μM) and 1:5 (5 μM) molar ratios. Data are represented as the mean of three independent experiments, and error bars represent the standard deviation.

Among all the evaluated compounds, **8e** and **8f** were able to reduce PrP conversion with a conversion percentage of 44 ± 10% and 27 ± 3%, respectively. In Figure 3, the seeding kinetics of PrP in the presence of **8e** and **8f** is shown to be inhibited. Moreover, the shortening of the lag phase of the curve is observed for both compounds when compared to the control and J8 curve (Figure 3A). The reduction was concentration-dependent: **8e** achieved a more than 50% decrease in PrP conversion at 10 μM, while **8f** exhibited the same potency at 20 μM (Figure 3B). **8f** was more efficient than **8e**, mainly at low concentrations (Figure 4B).

The aggregates produced in the presence of the compounds in the RT-QuIC experiment presented different conversion kinetics with varying curve profiles, including shortening in their lag phases. This could alter the final aggregates formed in terms of structure and toxicity. For this reason, these aggregates were compared in a cell viability assay using N2a cells. Although the compounds were shown to accelerate the lag phase and the exponential phase constant rate, their toxicity to N2a cells was comparable to the control (see Supporting Information File).

Protein aggregation can occur via different routes and form highly polymorphic species with other secondary structures and stability [35]. The environmental conditions, pH, temperature, agitation, ionic strength, and denaturant concentration are vital factors in generating variability [36,37]. An increase in temperature leads to PrP thermal denaturation, with solvent-exposed nonpolar residues, and consequently, aggregation [38,39]. Here, we chose to observe PrP aggregation at 60 °C (Figure 4) to investigate the effect of the compounds **8e** and **8f** on aggregates formed under conditions different than those presented above.

**Figure 5:**
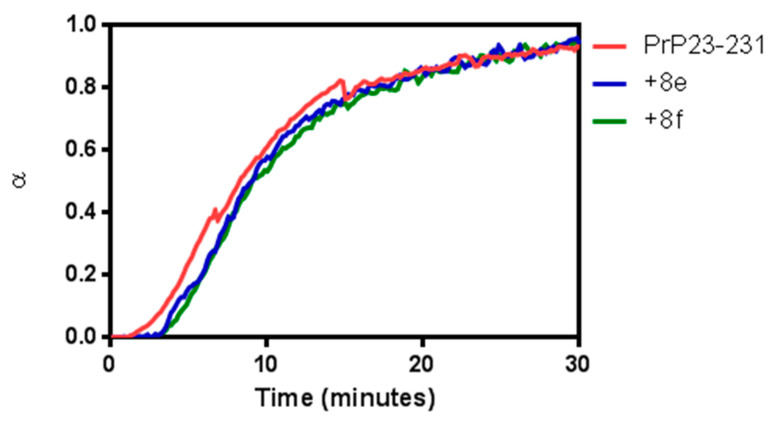
Representation of the fraction aggregated/non-aggregated (α) of PrP23-231 samples. PrP23-231 (1 μM) was incubated at 60 °C in the absence (red line) or presence of compounds (1 μM), and light scattering (LS) was monitored over time. LS increase was analyzed relative to initial LS values at 25 °C and normalized to the final values of each curve. Representative data of three independent experiments.

The temperature chosen for this assay induced the formation of aggregates with less ThT binding compared to lower temperatures (35–45 °C) [38]. PrP23-231 light scattering increased at 60 °C, reaching a plateau within 30 min, confirming PrP23-231 aggregation under this condition (Figure 4A). The addition of **8e** and **8f** decreased aggregation (Figure 4A) without changing its kinetics (better visualized in Figure 5). This effect is concentration-dependent, reaching a maximum effect at a 1:5 (PrP: compound) molar ratio (Figure 4B), as observed in the propagation experiments. The data suggest that **8e** and **8f** can modulate the aggregation of varied types of PrP aggregates.

**Figure 6:**
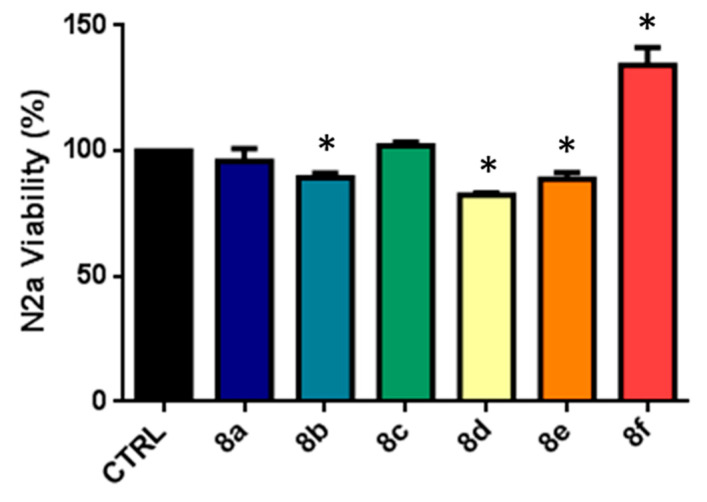
N2a cell line viability in the presence of 10 µM of compounds **8a–f**. Cells were treated with the compounds for 48 h, and the cell viability was measured through the MTT method. This set of compounds does not promote an inhibitory effect on N2a cell viability at 10 µM. * *p* > 0.005; *n* = 3.

The compounds must present low toxicity to neural cells as a first-order requisite of PrP conversion inhibitors to treat PrP diseases. Thus, our assay was designed to evaluate the cytotoxicity of compounds **8e** and **8f** on the N2a cell line, a neural cell line frequently used in PrP conversion studies. Figure 6 shows that none of the compounds used in this assay negatively affected cell viability at the concentration of 10 µM. More specifically, **8e** demonstrated very little inhibition of N2a cell line viability among the compounds with more prominent effects, and **8f** appeared to induce cell proliferation in the concentration used.

## 3. Conclusions

In conclusion, eight 1-alkyl-6-amino-4-quinolone derivatives (**5a–d** and **6a–d**) were synthesized and used in the preparation of six 2,5-dichloro-3,6-bis((1-alkyl-4-quinolone-6-yl)amino)-1,4-benzoquinone derivatives (**8a**–**f**), in a regioselective way. These compounds were evaluated regarding their prion conversion inhibition ability, fibrillation modulatory effect, and anti-aggregative profile. The results shown in this work indicate that compounds **8e** and **8f** are promising new candidates to be used as lead compounds for developing new PrP inhibitors. Further analyses are required to understand the mechanisms of PrP conversion inhibition and confirm their in vivo effects.

## 4. Materials and Methods

### 4.1. Synthesis

#### 4.1.1. General

All reagents and solvents were purchased from Merck & Co and used without further purification. Melting points were measured with a Fisher-Johns apparatus. NMR spectra were recorded on a Varian spectrometer operating at 500.00 MHz or 300.00 MHz (^1^H) and 125.00 MHz or 75.00 MHz (^13^C) using DMSO-*d*_6_ or CDCl_3_ as a solvent. Chemical shifts were reported in parts per million (ppm) relative to the solvent. Hydrogen and carbon NMR spectra were typically obtained at room temperature. The two-dimensional experiments were conducted using standard Varian Associates automated data acquisition and processing programs. Multiplicities were abbreviated as follows: s for singlets, d for doublets, t for triplets, q for quartets, quin for quintets, sxt for sextets, dd for doublet of doublets, m for multiplets, tt for a triplet of triplets, and br for broad signals. IR spectra were recorded on an ABB FTLA2000 spectrophotometer (KBr pellets) in the range of 4000–400 cm^−1^. The synthetic methods for preparing substances **4a**–**d**, **5a**–**d** [27,30], and **6a** [30], as well as their structural characterization, have already been reported.

#### 4.1.2. Ester Hydrolysis: General Procedure

First, 1 mmol of the corresponding ethyl 1-alkyl-6-amino-4-oxo-1,4-dihydroquinoline-3-carboxylate (**4b–d**), 12.5 mmol (500 mg) of sodium hydroxide, and 15 mL of ethanol were added to a round-bottom flask and kept under stirring at room temperature for 24 h, upon which chromatographic analysis showed complete consumption of the starting material (CH_2_Cl_2_/MeOH 20:1 as eluent). The solvent was evaporated, and the resulting residue was dissolved in 15 mL of distilled water. Neutralization with a dropwise addition of concentrated hydrochloric acid until pH 5–6 provided the product as a precipitated solid, which was then filtrated, washed with water, and recrystallized from ethanol.

6-amino-4-oxo-1-propyl-1,4-dihydroquinoline-3-carboxylic acid (**6b**). 45% yield; yellow solid; m.p. 242–244 °C. IV ν_max_ (cm^−1^, KBr): 3436, 3334, 1706, 1611, 1420. ^1^H NMR (500.00 MHz, DMSO-*d*_6_-*δ* 2.50 ppm), *δ* in ppm: 8.73 (s, 1H, H-2), 7.74 (d, 1H, *J* = 9.2 Hz, H-8), 7.46 (d, 1H, *J* = 2.7 Hz, H-5), 7.24 (dd, 1H, *J* = 9.2, 2.7 Hz, H-7), 5.72 (s, 2H, NH_2_), 4.43 (t, 2H, *J* = 7.4 Hz, H-1′), 1.75 (sxt, 2H, *J* = 7.4 Hz, H-2′), 0.91 (t, 3H, *J* = 7.4 Hz, H-3′). ^13^C-APT NMR (125.00 MHz, DMSO-*d*_6_-*δ* 39.51 ppm), *δ* in ppm: 176.74 (C-4); 166.74 (CO_2_H), 147.47 (C-6 or C-4a), 145.31 (C-2), 130.53 (C-8a), 127.19 (C-6 or C-4a), 122.51 (C-7), 118.87 (C-8), 105.82 (C-3), 105.51 (C-5), 54.98 (C-1′), 22.04 (C-2′), 10.34 (C-3′). HRMS (ESI) *m*/*z* calcd for C_13_H_15_N_2_O_3_^+^ [M + H]^+^: 247.1077, found 247.1077.

6-amino-4-oxo-1-pentyl-1,4-dihydroquinoline-3-carboxylic acid (**6c**). 51% yield; yellow solid; m.p. 214–216 °C. IR ν_max_ (cm^−1^, KBr): 3435, 3340, 1708, 1604, 1419. ^1^H NMR (500.00 MHz, DMSO-*d*_6_-*δ* 2.50 ppm), *δ* in ppm: 8.73 (s, 1H, H-2), 7.73 (d, 1H, *J* = 9.2 Hz, H-8), 7.46 (d, 1H, *J* = 2.7 Hz, H-5), 7.24 (dd, 1H, *J* = 9.2, 2.7 Hz, H-7), 5.72 (s, 2H, NH_2_), 4.46 (t, 2H, *J* = 7.4 Hz, H-1′), 1.80 (sxt, 2H, *J* = 7.2 Hz, H-2′), 1.35–1.28 (m, 4H, H-3′ e H-4′), 0.86 (t, 3H, *J* = 7.2 Hz, H-5′). ^13^C-APT NMR (75.00 MHz, DMSO-*d*_6_-δ 39.51 ppm), δ in ppm: 176.64 (C-4); 166.56 (CO_2_H), 147.40 (C-6 or C-4a), 145.18 (C-2), 130.39 (C-8a), 127.16 (C-6 or C-4a), 122.38 (C-7), 118.73 (C-8), 105.83 (C-3), 105.44 (C-5), 53.48 (C-1′), 28.32 (C-2′), 27.72 (C-3′ or C-4′), 21.38 (C-3′ or C-4′), 10.34 (C-5′). HRMS (ESI) *m*/*z* calcd for C_15_H_19_N_2_O_3_^+^ [M + H]^+^: 275.1390, found 275.1390.

6-amino-1-benzyl-4-oxo-1,4-dihydroquinoline-3-carboxylic acid (**6d**). 49% yield; yellow solid; m. p. 275–278 °C. IV ν_max_ (cm^−1^, KBr): 3445, 3357, 1695, 1605, 1432. ^1^H NMR (300.00 MHz, DMSO-*d*_6_-*δ* 2.50 ppm), *δ* in ppm: 8.93 (s, 1H, H-2), 7.60 (d, 1H, *J* = 9.2 Hz, H-8), 7.45 (d, 1H, *J* = 2.7 Hz, H-5), 7.39–7.29 (m, 3H, H-3″/5″ and H-4″), 7.24 (dd, 2H, *J* = 8.1 and 1.6 Hz, H-2″/6″), 5.75 (s, 2H, H-1′), 5.69 (s, 1H, NH_2_). ^13^C-APT NMR (75.00 MHz, DMSO-*d*_6_-δ 39.51 ppm), δ in ppm: 177.27 (C-4); 167.12 (CO_2_H), 147.72 (C-6 or C-4a), 146.30 (C-2), 135.46 (C-1″), 131.05 (C-8a), 129.10 (C-3″/5″), 128.29 (C-4″), 127.53 (C-6 or C-4a), 126.77 (C-2″/6″), 122.73 (C-7), 119.65 (C-8), 106.44 (C-3), 105.80 (C-5), 56.85 (C-1′). HRMS (ESI) *m*/*z* calcd for C_17_H_15_N_2_O_3_^+^ [M + H]^+^: 295.1077, found 295.1077.

#### 4.1.3. Dimerization through Benzoquinone Amination: General Procedure

In a round-bottom flask containing 0.163 mmol (40.0 mg) of p-chloranil (**7**) solubilized in 5.0 mL of ethanol, a previously prepared solution of 0.325 mmol of the respective 6-amino-4-quinolone in 5.0 mL of ethanol was added dropwise at room temperature under constant stirring. The mixture was kept under stirring at 60 °C for 24 h when another 0.163 mmol (40.0 mg) of p-chloranil (**7**) was added to the reaction flask, which was kept at this temperature for another 24 h. At the end of the reaction, the precipitated solid was isolated by filtration and washed with 30.0 mL of heated ethanol. The product was dried in a vacuum-sealed desiccator.

2,5-dichloro-3,6-bis((3-(ethoxycarbonyl)-1-ethyl-4-oxo-1,4-dihydroquinolin-6-yl)amino)-1,4-benzoquinone (**8a**): 83% yield, dark brown solid, m.p. > 300 °C. IR ν_max_ (cm^−1^, KBr): 3222, 1724, 1588, 1509, 804. ^1^H NMR (500.00 MHz, DMSO-*d*_6_-δ 2.50 ppm), δ in ppm: 9.78 (s, 1H, NH), 8.64 (s, 1H, H-2), 7.94 (d, 1H, *J* = 2.6 Hz, H-5), 7.80 (d, 1H, *J* = 9.1 Hz, H-8), 7.64 (dd, 1H, *J* = 9.1 and 2.6 Hz, H-7), 4.42 (q, 2H, *J* = 7.1 Hz, H-1′), 4.24 (q, 2H, *J* = 7.1 Hz, CO_2_CH_2_CH_3_), 1.41 (t, 3H, *J* = 7.1 Hz, H-2′), 1.30 (t, 3H, *J* = 7.1 Hz, CO_2_CH_2_CH_3_). ^13^C NMR (75.00 MHz, with 4 eq. of K_2_CO_3_ in DMSO-*d*_6_-*δ* 39.51 ppm –) *δ* in ppm: 172.46 (C-1″/4″), 172.22 (both C-4), 164.54 (both CO_2_Et), 147.09 (both C-2), 145.95, 145.52, 143.74, 128.36, 120.44, 117.43, 116.36, 109.23 (both C-3), 107.59 (C-2″/5″ or C-3″/6″), 59.26 (both CO_2_CH_2_CH_3_), 47.68 (both C-1′), 14.03 (both C-2′ or both CO_2_CH_2_CH_3_), 13.95 (both CO_2_CH_2_CH_3_ or both C-2′). HRMS (ESI) *m*/*z* calcd for C_34_H_30_Cl_2_N_4_NaO_8_^+^ [M + Na]^+^: 715.1333, found 715.1331 and calcd for C_34_H_31_Cl_2_N_4_O_8_^+^ [M + H]^+^: 693.1513, found 693.1511.

2,5-dichloro-3,6-bis((3-(ethoxycarbonyl)-4-oxo-1-propyl-1,4-dihydroquinolin-6-yl)amino)-1,4-benzoquinone (**8b**): 56% yield, dark brown solid, m.p. > 300 °C. IR ν_max_ (cm^−1^, KBr): 3228, 2979, 1729, 1589, 1505, 805. ^1^H NMR (500.00 MHz, DMSO-*d*_6_-δ 2.50 ppm), *δ* in ppm: 9.78 (s, 2H, both NH), 8.63 (s, 2H, both H-2), 7.94 (d, 2H, *J* = 2.7 Hz, both H-5), 7.80 (d, 2H, *J* = 9.0 Hz, both H-8), 7.61 (dd, 2H, *J* = 9.0 and 2.7 Hz, both H-7), 4.34 (t, 4H, *J* = 7.3 Hz, both H-1′ sets), 4.24 (q, 4H, *J* = 7.1 Hz, both CO_2_CH_2_CH_3_ sets), 1.82 (sxt, 4H, *J* = 7.3 Hz, both H-2′ sets), 1.30 (t, 3H, *J* = 7.1 Hz, both CO_2_CH_2_CH_3_ sets), 0.94 (t, 6H, *J* = 7.3 Hz, both H-3′ sets). ^13^C-APT NMR (75.00 MHz, DMSO-*d*_6_—δ 39.51 ppm) δ in ppm: 173.75 (C-1″/4″), 172.26 (both C-4), 164.51 (both CO_2_Et), 148.65 (both C-2), 142.53, 135.88 (both C-8a), 134.99 (both C-4a or both C-6), 128.96 (both C-7), 127.88, 120.68 (both C-5), 116.97 (both C-8), 109.54 (both C-3), 105.94 (C-2″/5″ or C-3″/6″), 59.55 (both CO_2_CH_2_CH_3_), 54.05 (both C-1′), 21.78 (both C-2′), 14.16 (both CO_2_CH_2_CH_3_), 10.43 (both C-3′). HRMS (ESI) *m*/*z* calcd for C_36_H_34_Cl_2_N_4_NaO_8_^+^ [M + Na]^+^: 743.1646, found 743.1643 and calcd for C_36_H_35_Cl_2_N_4_O_8_^+^ [M + H]^+^: 721.1826, found 721.1823.

2,5-dichloro-3,6-bis((3-(ethoxycarbonyl)-4-oxo-1-pentyl-1,4-dihydroquinolin-6-yl)amino)-1,4-benzoquinone (**8c**): 41% yield, dark brown solid, m. p. > 300 °C. IR ν_max_ (cm^−1^, KBr): 3241, 2957, 1721, 1590, 1503, 805. ^1^H NMR (500.00 MHz, DMSO-*d*_6_-*δ* 2.50 ppm), *δ* in ppm: 9.77 (s, 2H, both NH), 8.62 (s, 2H, both H-2), 7.94 (d, 2H, *J* = 2.9 Hz, both H-5), 7.78 (d, 2H, *J* = 9.3 Hz, both H-8), 7.63 (dd, 2H, *J* = 9.3 and 2.9 Hz, both H-7), 4.36 (q, 4H, *J* = 7.1 Hz, both H-1′ sets), 4.24 (q, 4H, *J* = 7.3 Hz, both CO_2_CH_2_CH_3_ sets), 1.79 (quin, 4H, *J* = 7.1 Hz, both H-2′ sets), 1.37–1.31 (m, 8H, both H-3′ and H-4′ sets), 1.30 (t, 3H, *J* = 7.3 Hz, both CO_2_CH_2_CH_3_ sets), 0.87 (t, 6H, *J* = 7.3 Hz, both H-5′ sets). ^13^C-APT NMR (75.00 MHz, DMSO-*d*_6_-*δ* 39.51 ppm) *δ* in ppm: 173.58 (C-1″/4″), 172.19 (both C-4), 164.40 (both CO_2_Et), 148.33 (both C-2), 142.50, 135.90 (both C-8a), 134.95 (both C-4a or both C-6), 128.96 (both C-7), 127.88, 120.77 (both C-5), 116.80 (both C-8), 109.71 (both C-3), 105.96 (C-2″/5″ or C-3″/6″), 59.46 (both CO_2_CH_2_CH_3_), 52.61 (both C-1′), 27.99 (both C-2′), 27.79 (both C-3′ or both C-4′), 21.37 (both C-3′ or both C-4′), 14.04 (both CO_2_CH_2_CH_3_), 13.43 (both C-5′). HRMS (ESI) *m*/*z* calcd for C_40_H_42_Cl_2_N_4_NaO_8_^+^ [M + Na]^+^: 799.2272, found 799.2269.

2,5-dichloro-3,6-bis((3-carboxy-4-oxo-1-propyl-1,4-dihydroquinolin-6-yl)amino)-1,4-benzoquinone (**8d**): 57% yield, dark brown solid, m.p. 250 °C with decomposition. IR ν_max_ (cm^−1^, KBr): 3262, 1724, 1653, 1584, 821. ^1^H NMR (500.00 MHz, DMSO-*d*_6_-*δ* 2.50 ppm), *δ* in ppm: 9.79 (s, 2H, both NH), 8.96 (s, 2H, both H-2), 8.04 (d, 2H, *J* = 2.7 Hz, both H-5), 8.02 (d, 2H, *J* = 9.3 Hz, both H-8), 7.80 (dd, 2H, *J* = 9.3 and 2.7 Hz, both H-7), 4.53 (t, 4H, *J* = 7.7 Hz, both H-1′ sets), 1.88 (sxt, 4H, *J* = 7.7 Hz, both H-2′ sets), 0.96 (t, 6H, *J* = 7.7 Hz, both H-3′ sets). ^13^C-APT NMR (125.00 MHz, DMSO-*d*_6_-*δ* 39.51 ppm) *δ* in ppm: 177.12 (both C-4), 173.89 (C-1″/4″), 165.69 (both CO_2_H), 148.42 (both C-2), 142.16 (C-2″/5″ or C-3″/6″), 136.33 (both C-4a or both C-6), 136.04 (both C-8a), 130.02 (both C-7), 125.28 (both C-4a or both C-6), 118.98 (both C-5), 117.81 (both C-8), 107.68 (C-2″/5″ or C-3″/6″), 107.24 (both C-3), 54.94 (both C-1′), 21.91 (both C-2′), 10.25 (both C-3′). HRMS (ESI) *m*/*z* calcd for C_32_H_27_Cl_2_N_4_O_8_^+^ [M + H]^+^: 665.1200, found 665.1201.

2,5-dichloro-3,6-bis((3-carboxy-4-oxo-1-pentyl-1,4-dihydroquinolin-6-yl)amino)-1,4-benzoquinone (**8e**): 45% yield, dark brown solid, m. p. > 300 °C. IR ν_max_ (cm^−1^, KBr): 3257, 1722, 1654, 1583, 820. ^1^H NMR (500.00 MHz, DMSO-*d*_6_-*δ* 2.50 ppm), *δ* in ppm: 9.79 (s, 2H, both NH), 8.97 (s, 2H, both H-2), 8.05 (d, 2H, *J* = 2.7 Hz, both H-5), 8.01 (d, 2H, *J* = 9.3 Hz, both H-8), 7.80 (dd, 2H, *J* = 9.3 and 2.7 Hz, both H-7), 4.56 (t, 4H, *J* = 6.8 Hz, both H-1′ sets), 1.85 (quin, 4H, *J* = 6.8 Hz, both H-2′ sets), 1.39–1.31 (m, 8H, both H-3′ and H-4′ sets), 0.88 (t, 6H, *J* = 6.8 Hz, both H-5′ sets). ^13^C-APT NMR (75.00 MHz, DMSO-*d*_6_-*δ* 39.51 ppm) *δ* in ppm: 177.12 (both C-4), 173.88 (C-1″/4″), 165.70 (both CO_2_H), 148.29 (both C-2), 142.18 (C-2″/5″ or C-3″/6″), 136.32 (both C-4a or both C-6), 136.03 (both C-8a), 130.05 (both C-7), 125.30 (both C-4a or both C-6), 119.04 (both C-5), 117.78 (both C-8), 107.68 (C-2″/5″ or C-3″/6″), 107.31 (both C-3), 53.62 (both C-1′), 28.23 (both C-2′), 27.69 (both C-3′ or both C-4′), 21.36 (both C-3′ or both C-4′), 13.41 (both C-5′). HRMS (ESI) *m*/*z* calcd for C_36_H_35_Cl_2_N_4_O_8_^+^ [M + H]^+^: 721.1826, found 721.1826.

2,5-dichloro-3,6-bis((1-benzyl-3-carboxy-4-oxo-1,4-dihydroquinolin-6-yl)amino)-1,4-benzoquinone (**8f**): 66% yield, dark brown solid, m.p. > 300 °C. IR ν_max_ (cm^−1^, KBr): 3263, 1717, 1655, 1585, 819. ^1^H NMR (500.00 MHz, DMSO-*d*_6_-*δ* 2.50 ppm), *δ* in ppm: 9.89 (s, 2H, both NH), 9.22 (s, 2H, both H-2), 7.98 (d, 2H, *J* = 2.7 Hz, both H-5), 7.85 (d, 2H, *J* = 9.4 Hz, both H-8), 7.67 (dd, 2H, *J* = 9.4 and 2.7 Hz, both H-7), 7.40–7.28 (m, 10H, both H-2′/6′, H-3′/5′ and H-4′ sets), 5.86 (s, 4H, both CH_2_ sets). ^13^C-APT NMR (125.00 MHz, DMSO-*d*_6_-δ 39.51 ppm) δ in ppm: 178.05 (both C-4), 174.52 (C-1″/4″), 166.30 (both CO_2_H), 149.71 (both C-2), 142.78 (C-2″/5″ or C-3″/6″), 137.12 (both C-4a or both C-6), 136.92 (both C-8a), 135.66 (both C-1′), 130.55 (both C-7), 129.42 (both C-3′/5′), 128.60 (C-4′), 127.32 (both C-2′/6′), 126.14 (both C-4a or both C-6), 119.58 (both C-5), 118.94 (both C-8), 108.55 (C-2″/5″ or C-3″/6″), 108.34 (both C-3), 57.12 (both NCH_2_). HRMS (ESI) *m*/*z* calcd for C_40_H_27_Cl_2_N_4_O_8_^+^ [M + H]^+^: 761.1200, found 761.1199.

### 4.2. Biological Assays

#### 4.2.1. Prion Protein Expression and Purification

The murine recombinant full-length prion protein (PrP23-231) was expressed in Escherichia coli and purified by high-affinity chromatography according to the protocol described in [11].

#### 4.2.2. In Vitro-Produced Fibril Preparation and Amplification

PrP23-231 fibrils were prepared in denaturing conditions according to previous works [11,33]. Briefly, PrP23-231 was lyophilized after purification and resuspended in 6 M GdnHCl. This sample was diluted in a 10 mM phosphate buffer at pH 6.8 containing 1 M GdnHCl, 3 M urea, and 150 mM NaCl to a final concentration of 10 μM. After that, it was incubated for 70 h at 37 °C with continuous shaking at 600 rpm in conical plastic tubes in a 0.75 mL reaction volume. This procedure leads to the formation of amyloid fibrils. These in vitro-produced fibrils were used as seeds to amplify recombinant PrP23-231 as substrate in a modified RT-QuIC protocol [11,33]. Four percent of seeds were incubated with 100 μM of 10 mM phosphate buffer at pH 7.4 containing 300 mM NaCl, 10 μM thioflavin T (ThT), and 4 μM PrP23-231 substrate. The sample was loaded into a black 96-well plate with four replicates for each sample and then sealed and transferred to a FluoStar OMEGA microplate reader (BMG LabTech) with double orbital shaking (1 min shaking at 700 rpm double orbital/1 min resting) at 42 °C for ~40 h. ThT fluorescence was measured with excitation at 450 nm and emission at 480 nm every ~15 min. Compounds were at 1 μM, 5 μM, 10 μM, and 20 μM, and the vehicle’s final concentration corresponded to 0.02% of DMSO. The exact amount of DMSO was added to PrP23-231 and PrP23-231 + seed control samples. Data are represented as the percentage of fibril conversion, using the condition PrP23-231 + seed as 100% conversion.

#### 4.2.3. Spectroscopic Measurements

PrP light scattering was acquired using a PC1 spectrofluorometer (ISS, Champaign, IL, USA) in an “L” geometry (at 90° relative to the excitation light). The samples were illuminated at 320 nm with data acquisition at 320 nm for kinetic experiments and from 300 to 340 nm to collect light-scattering spectra. PrP23-231 aggregation was induced by the temperature at 60 °C and evaluated for 30 min after reaching equilibrium. PrP23-231 was diluted to a 4 mM final concentration in a 50 mM Tris buffer containing 100 mM NaCl at pH 7.4. Compounds were 1 μM for kinetic measurements and 1 μM and 5 μM for steady-state measurements. Once the compound vehicle was DMSO, the same amount of DMSO (0.02%) was added to the PrP23-231 sample. Data were represented related to PrP23-231 or PrP23-231 + compound light scattering at 25 °C. The fraction aggregated/non-aggregated was also shown for kinetic comparison.

#### 4.2.4. MTT Assays

Ninety-six-well plates were used, and cells were grown to a confluence between 80 and 90% at the time of the assay. After 24 h, the medium was changed, the compounds or aggregates were added, and the cells were treated for 48 h. Then, 3-(4,5- dimethylthiazol-2-yl)-2,5-diphenyltetrazolium bromide (MTT) at 0.5 mg/mL in PBS was added to the cells for 3 h. The resulting crystals were solubilized in DMSO, and the plate was analyzed using a SpectraMax Paradigm Multi-mode Microplate Reader (Molecular Devices) at 570 nm and 650nm. The values corresponding to the difference between the absorption at 650 nm and 570 nm were used to calculate cell viability.

## Figures and Tables

**Table 1:** Compound screening according to RT-QuIC percentage of PrP conversion ± Standard error.

Compound	% Conversion
**5a**	109 ± 22
**5b**	118 ± 13
**5c**	90 ± 14
**5d**	100 ± 15
**6a**	102 ± 3
**6b**	73 ± 24
**6c**	94 ± 5
**6d**	83 ± 9
**8a**	97 ± 12
**8b**	78 ± 7
**8c**	113 ± 10
**8d**	62 ± 4
**8e**	44 ± 10
**8f**	27 ± 3

## Data Availability

Not applicable.

## Supplementary Materials

The following supporting information can be downloaded at: https://www.mdpi.com/article/10.3390/molecules27227935/s1, Figures S1–S51: Spectroscopic data.

## Sample Availability

Samples of compounds **5**, **6,** and **8** are available from the authors.
