# Supplementary material for: Aminoquinolones and Their Benzoquinone Dimer Hybrids as Modulators of Prion Protein Conversion
Source: Molecules. 2022 Nov 16;27(22):7935. doi: 10.3390/molecules27227935 (PMC9693643; doi:10.3390/molecules27227935)
Supplement: Supplementary file 1 [file molecules-27-07935-s001.zip › molecules-1967301-supplementary.pdf]

molecules

MDPI

Aminoquinolones and Their Benzoquinone Dimer Hybrids as Modulators of Prion Protein Conversion

Amanda Rodrigues Pinto Costa [1], Marcelly Muxfeldt [2], Fernanda da Costa Santos Boechat [1], Maria Cecília Bastos Vieira de Souza [1], Jerson Lima Silva [3], Marcela Cristina de Moraes [1], Luciana Pereira Rangel [2], Tuane Cristine Ramos Gonçalves Vieira [3] and Pedro Netto Batalha [1,*]

1 Instituto de Química, Universidade Federal Fluminense, Niterói 24020-141, RJ, Brazil
2 Faculdade de Farmácia, Universidade Federal do Rio de Janeiro, Rio de Janeiro 21941-902, RJ, Brazil
3 Instituto de Bioquímica Médica Leopoldo de Meis, Instituto Nacional de Ciência e Tecnologia de Biologia Estrutural e Bioimagem, Universidade Federal do Rio de Janeiro, Rio de Janeiro 21941-902, RJ, Brazil
* Correspondence: pedrobatalha@id.uff.br

Supporting Information

I) MR spectra..3

I. A) 6-amino-4-oxo-1-propyl-1,4-dihydroquinoline-3-carboxylic acid (6b) 4

I. B) 6-amino-4-oxo-1-pentyl-1,4-dihydroquinoline-3-carboxylic acid (6c) 6

I. C) 6-amino-1-benzyl-4-oxo-1,4-dihydroquinoline-3-carboxylic acid (6d) 9

I. 2,5-dichloro-3,6-bis((3-(ethoxycarbonyl)-1-ethyl-4-oxo-1,4-dihydroquinolin-6-yl)amino)-1,4-benzoquinone (8a) 12

I.E) 2,5-dichloro-3,6-bis((3-(ethoxycarbonyl)-4-oxo-1-propyl-1,4-dihydroquinolin-6-yl)amino)-1,4-benzoquinone (8b) 14

I.F) 2,5-dichloro-3,6-bis((3-(ethoxycarbonyl)-4-oxo-1-pentyl-1,4-dihydroquinolin-6-yl)amino)-1,4-benzoquinone (8c)... 17

I.G) 2,5-dichloro-3,6-bis((3-carboxy-4-oxo-1-propyl-1,4-dihydroquinolin-6-yl)amino)-1,4-benzoquinone (8d) 20

I.H) 2,5-dichloro-3,6-bis((3-carboxy-4-oxo-1-pentyl-1,4-dihydroquinolin-6-yl)amino)-1,4-benzoquinone (8e) 23

I.I) 2,5-dichloro-3,6-bis((1-benzyl-3-carboxy-4-oxo-1,4-dihydroquinolin-6-yl)amino)-1,4-benzoquinone (8f) 26

II) 2a cells cell viability assay in the presence of aggregates produced in RT-QuIC assays 29

I) NMR spectra

Through the analysis of the NMR spectra, significant differences were observed regarding the hydrogen and carbon chemical shifts associated with substances **8** compared to their 6-amino-4-quinolone precursors. In the ^{1}H NMR spectrum of substance **8d**, for example, both chemically equivalent NH appeared as a singlet at 9.79 ppm, more unshielded than the corresponding primary amino group (NH_2) present in its respective precursor **6b** (δ: 5.72 ppm), which can be explained by the conjugation of the amino group to the 3,6-dichloro-1,4-benzoquinone core. The two doublets at 8.04 (*J* = 2.7 Hz) and 8.02 (*J* = 9.3 Hz) ppm were assigned to the two sets of H-5 and H-8, respectively. Contrary to what was observed in precursor **6b**, H-5 was more unshielded than H-8 in dimer **8d**, which is evidence that the reaction was successful. H-2 and H-7, in turn, were associated with the singlet and doublet of doublet (*J* = 9.3 and 2.7 Hz) at 8.96 and 7.80 ppm, respectively. The other signals related to the *N*-propyl group presented chemical shift patterns and multiplicities like those observed in the ^{1}H NMR spectrum of precursor **6b**.

The analysis of substances **8** ^{1}H NMR spectra alone did not allow us to unequivocally confirm the regioselectivity and the success of the dimerization reaction. The parallel analysis of their ^{13}C-APT and HMBC spectra (Figure 3) allowed us to establish the occurrence of dimerization from the establishment of C-N bonds at positions 2 and 5 of the 1,4-benzoquinone core, in a regioselective manner, due to the presence of only one signal associated with chemically equivalent carbonyls at C-1'' and C-4'', with a three bond correlation to NH, in the HMBC spectra. The other carbons were assigned as described for precursors **5** and **6**. Figure S1 highlights the main HMBC correlations observed for **8d** as a representative example.

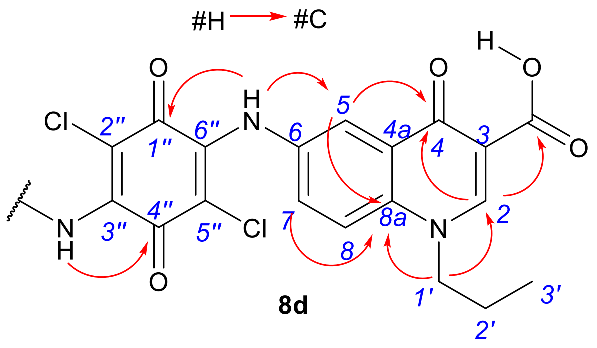

8d

Figure S1. Main HMBC correlations were observed for substances **8**, exemplified for **8d**.

I.A) 6-amino-4-oxo-1-propyl-1,4-dihydroquinoline-3-carboxylic acid (6b)

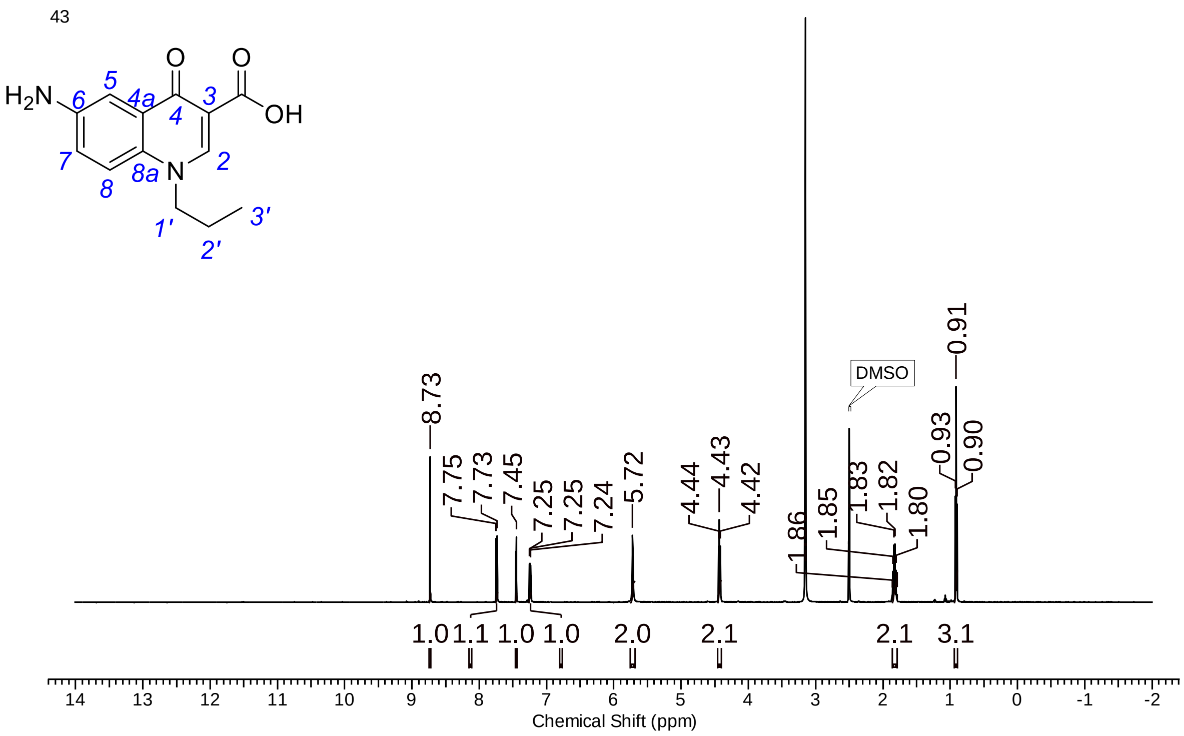

Figure S2. ^{1}H NMR spectrum of derivative **6b** (DMSO-*d*6, 500.00 MHz).

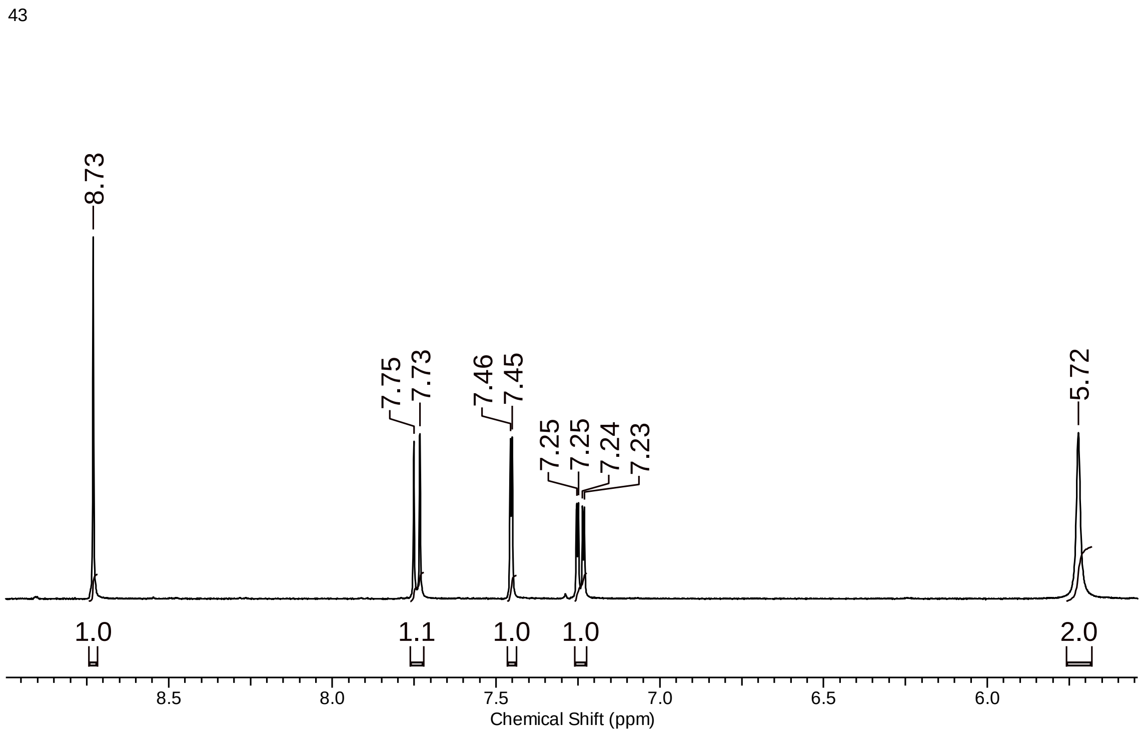

Figure S3. Expansion of the ^{1}H NMR spectrum of derivative **6b** (DMSO-*d*6, 500.00 MHz).

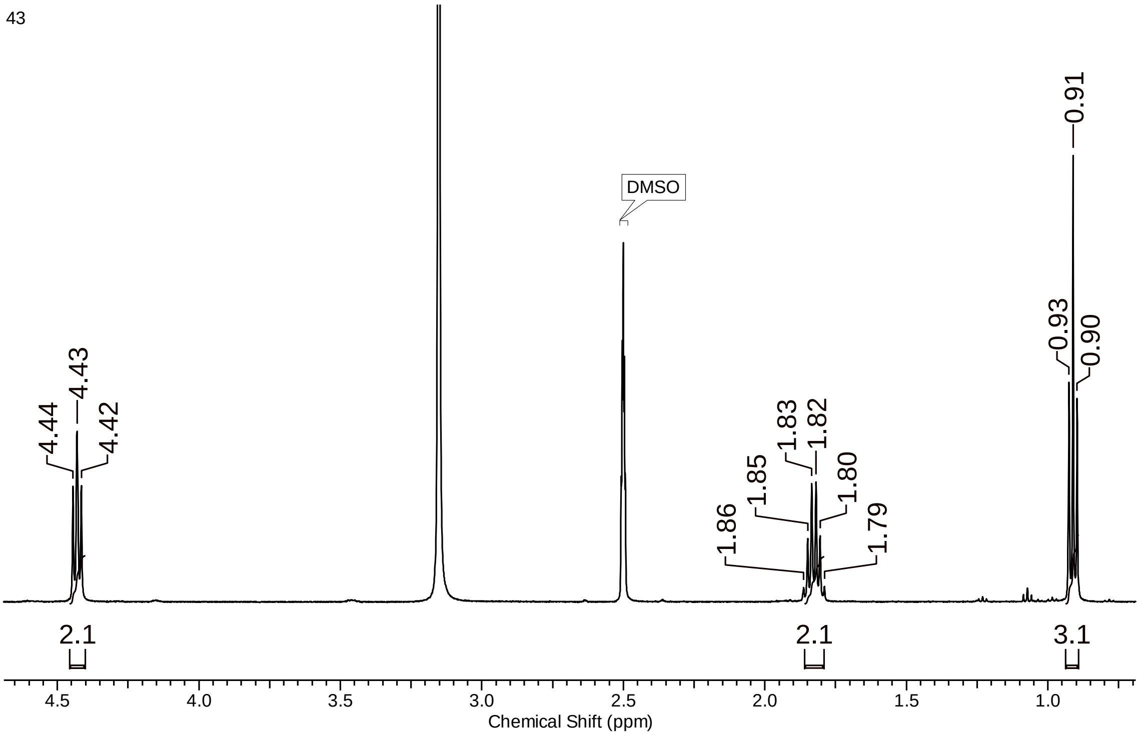

Figure S4. Expansion of the ^{1}H NMR spectrum of derivative **6b** (DMSO-d_6, 500.00 MHz).

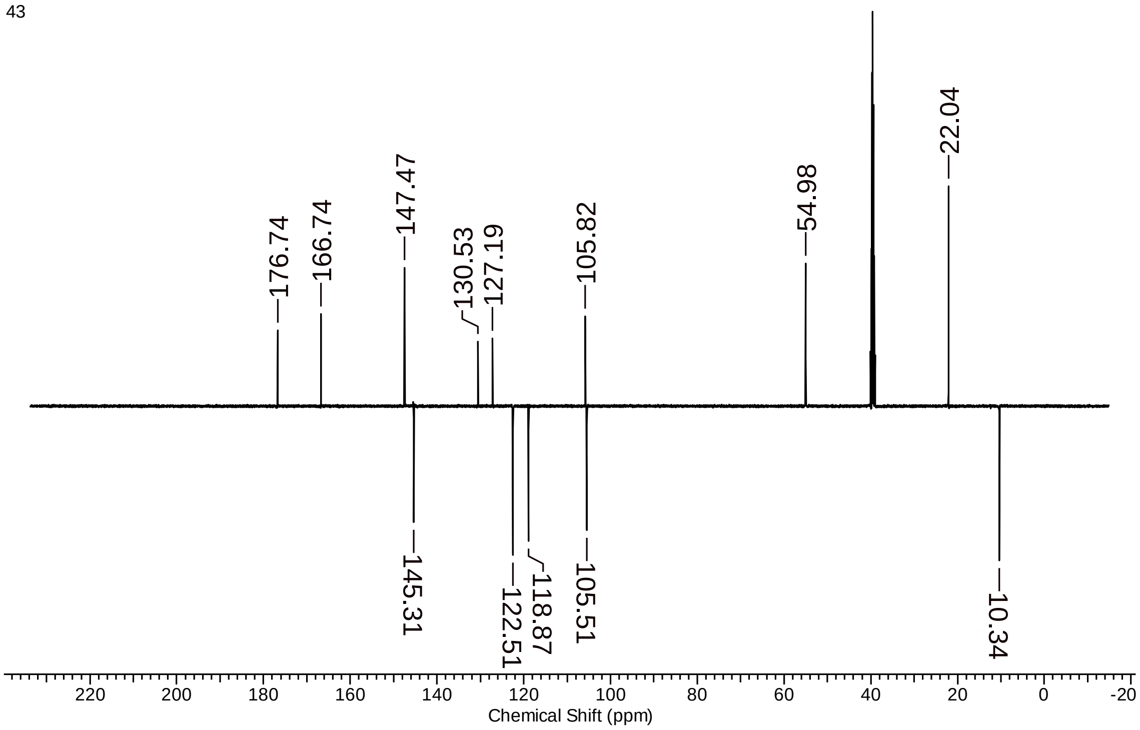

Figure S5. ^{13}C-APT NMR spectrum of derivative **6b** (DMSO-d_6, 125.00 MHz).

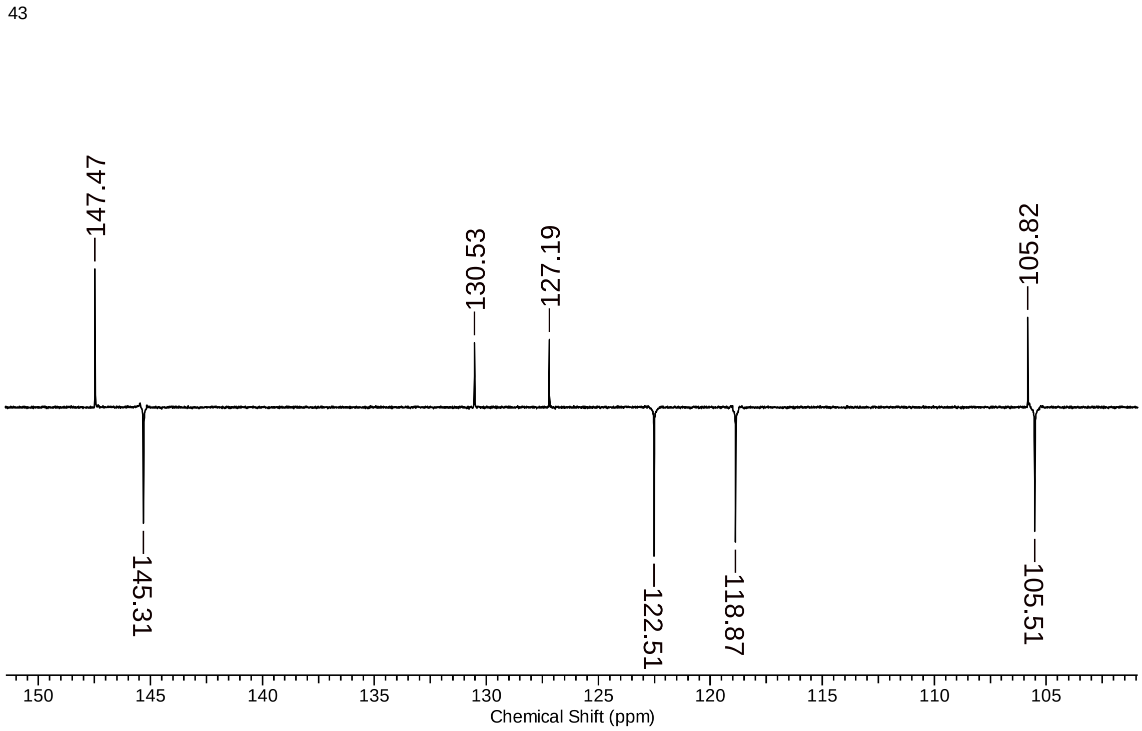

Figure S6. Expansion of the ^{13}C-APT NMR spectrum of derivative **6b** (DMSO-d_6, 125.00 MHz).

I.B) 6-amino-4-oxo-1-pentyl-1,4-dihydroquinoline-3-carboxylic acid (6c)

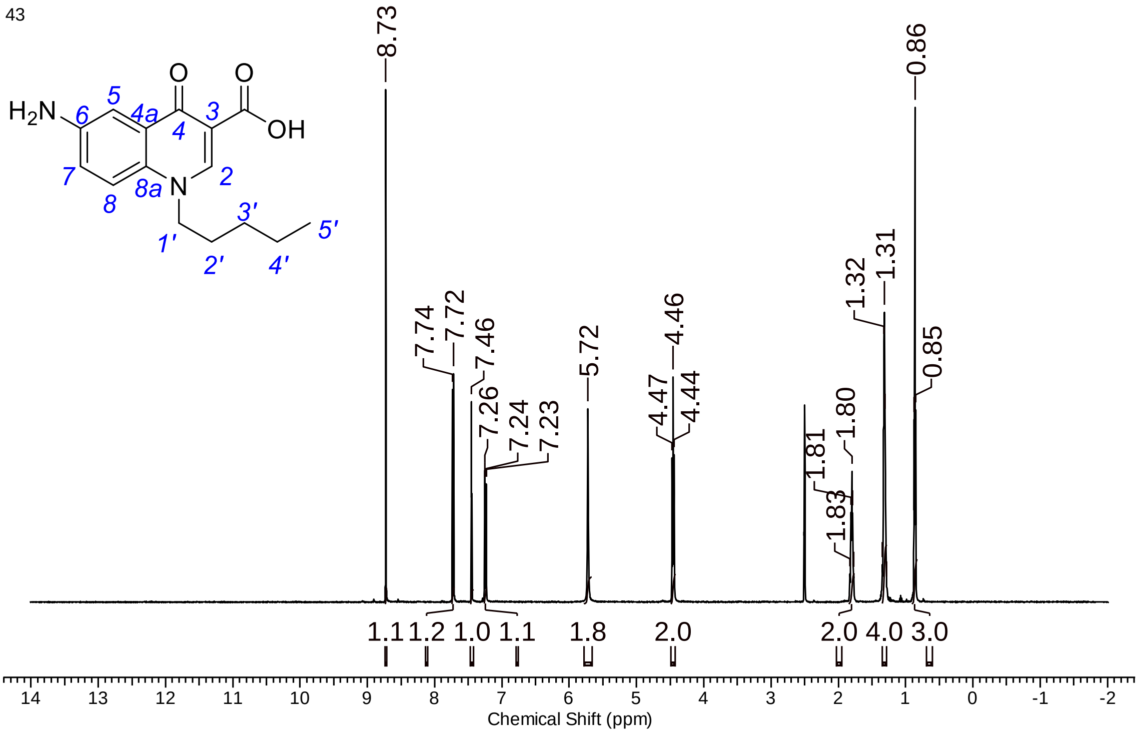

Figure S7. ^{1}H NMR spectrum of derivative **6c** (DMSO-d_6, 500.00 MHz).

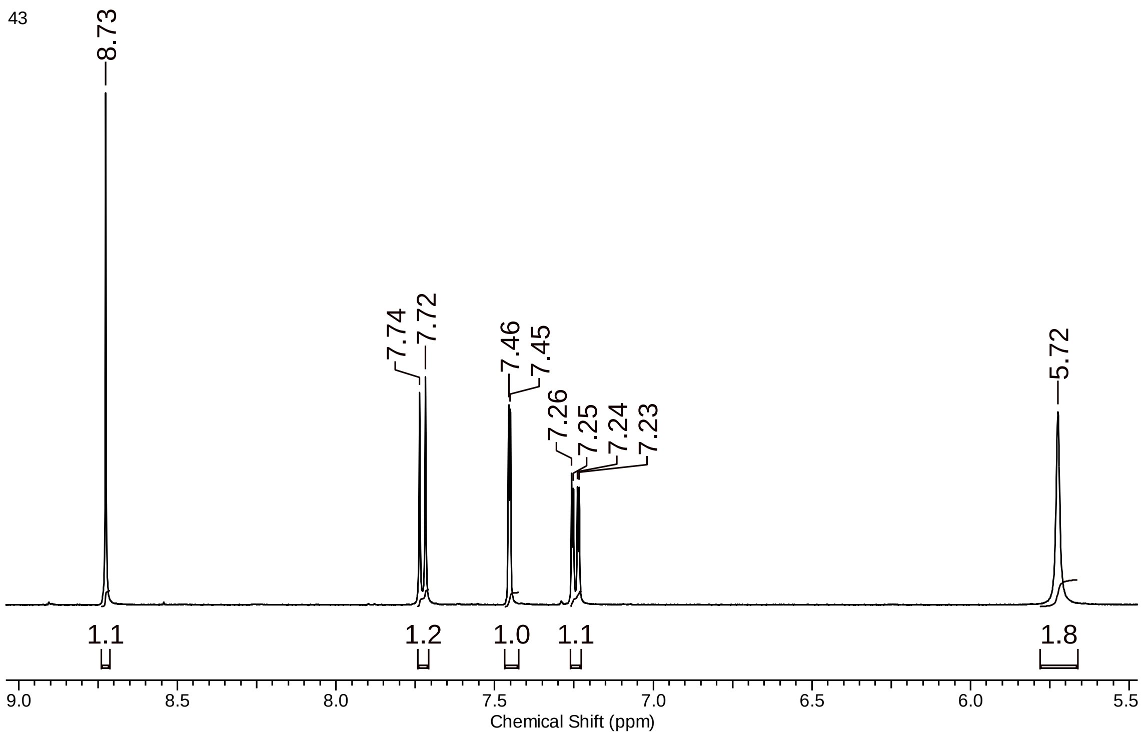

Figure S8. Expansion of the ^{1}H NMR spectrum of derivative **6c** (DMSO-d_6, 500.00 MHz).

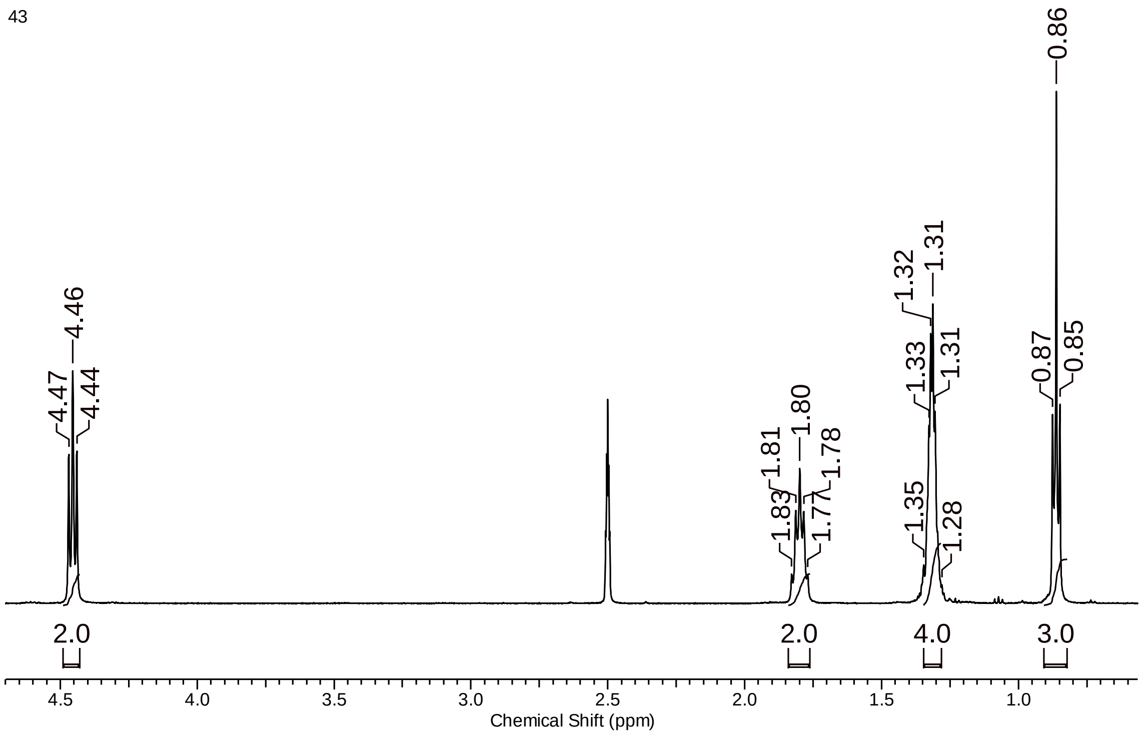

Figure S9. Expansion of the ^{1}H NMR spectrum of derivative **6c** (DMSO-d_6, 500.00 MHz).

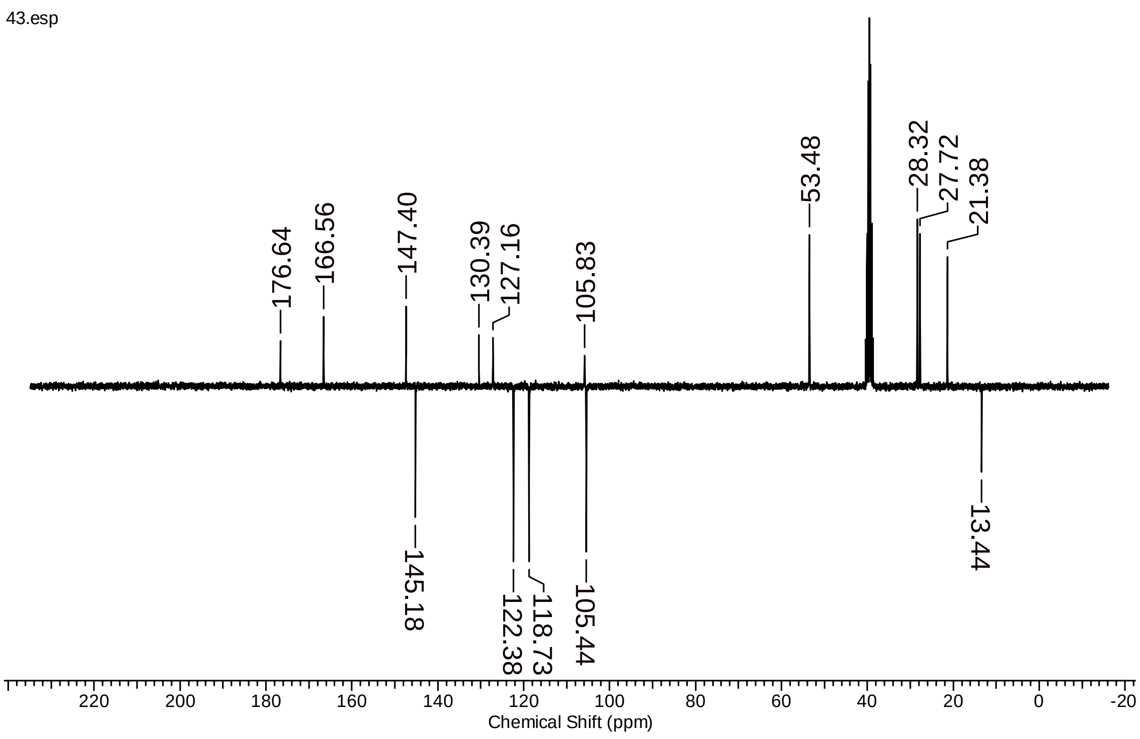

Figure S10. ^{13}C-APT NMR spectrum of derivative **6c** (DMSO-d_6, 75.00 MHz).

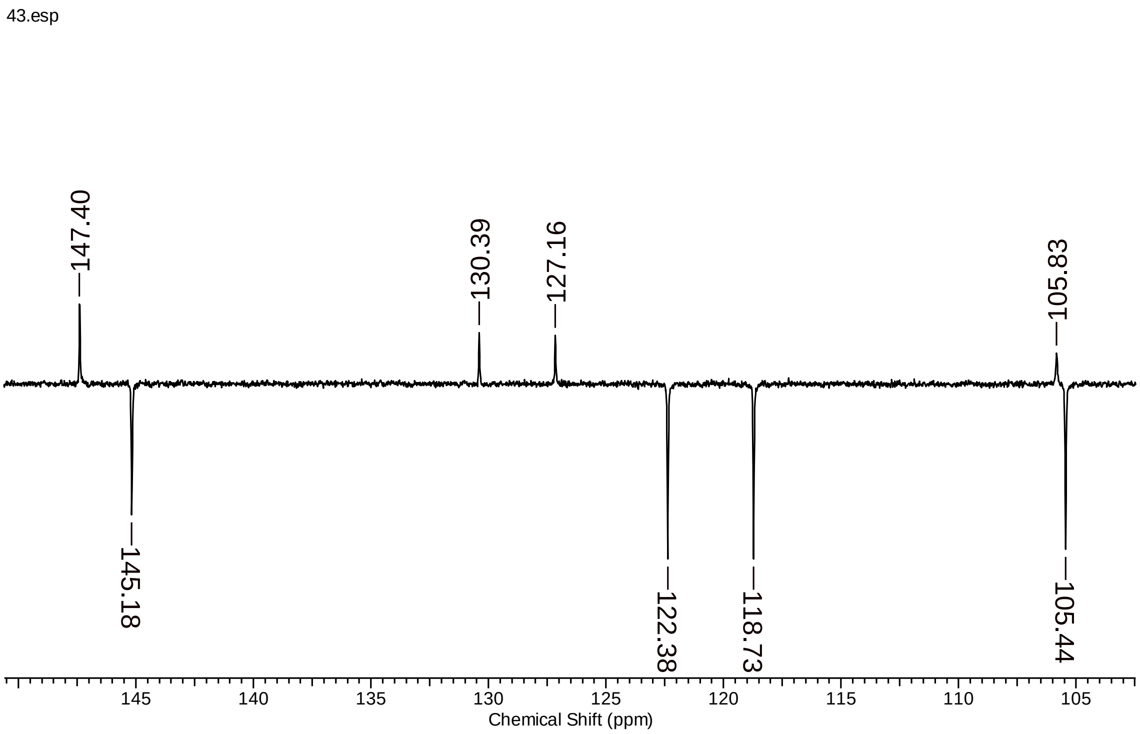

Figure S11. Expansion of the ^{13}C-APT NMR spectrum of derivative **6c** (DMSO-d_6, 75.00 MHz).

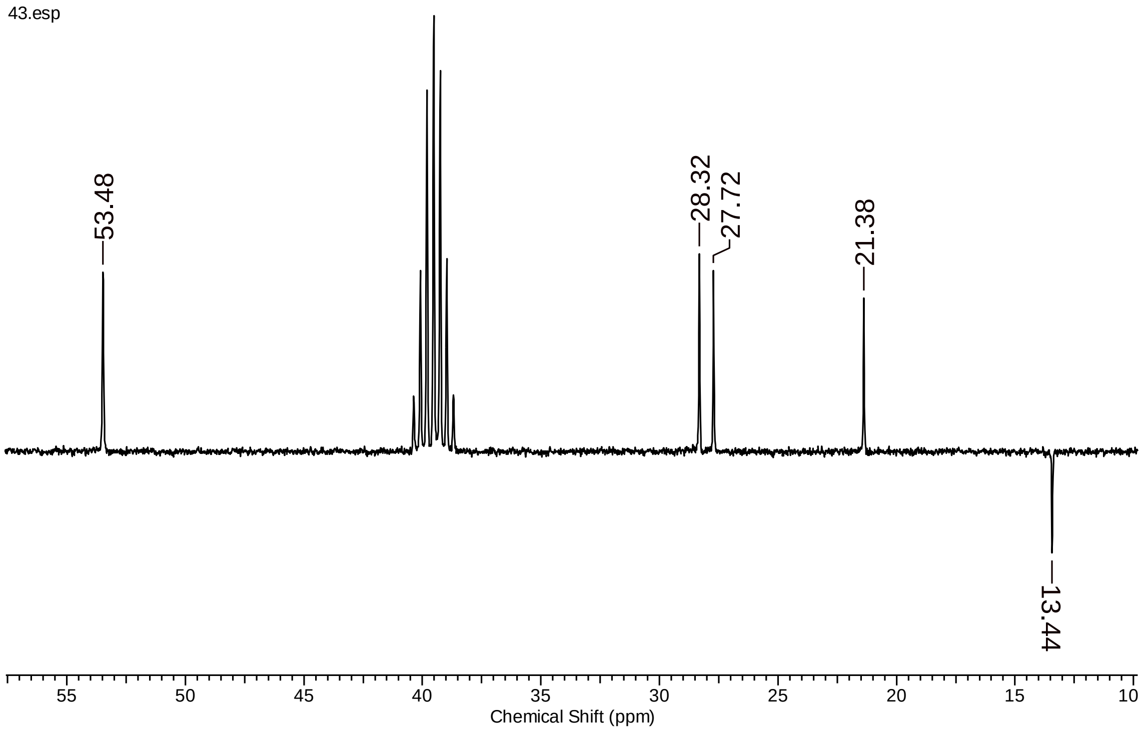

Figure S12. Expansion of the ^{13}C-APT NMR spectrum of derivative **6c** (DMSO-d_6, 75.00 MHz).

I.C) 6-amino-1-benzyl-4-oxo-1,4-dihydroquinoline-3-carboxylic acid (6d)

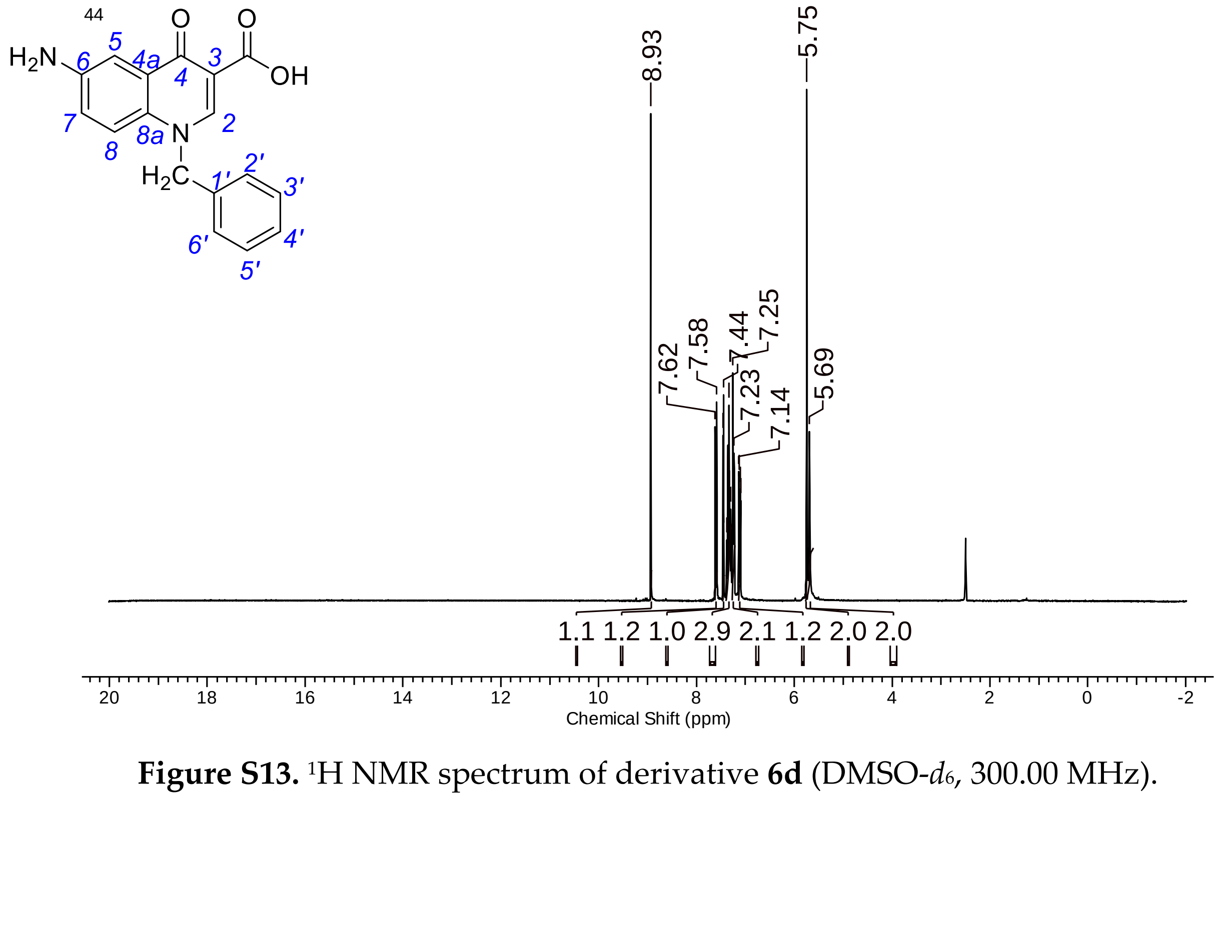

Figure S13. ^{1}H NMR spectrum of derivative **6d** (DMSO-d_6, 300.00 MHz).

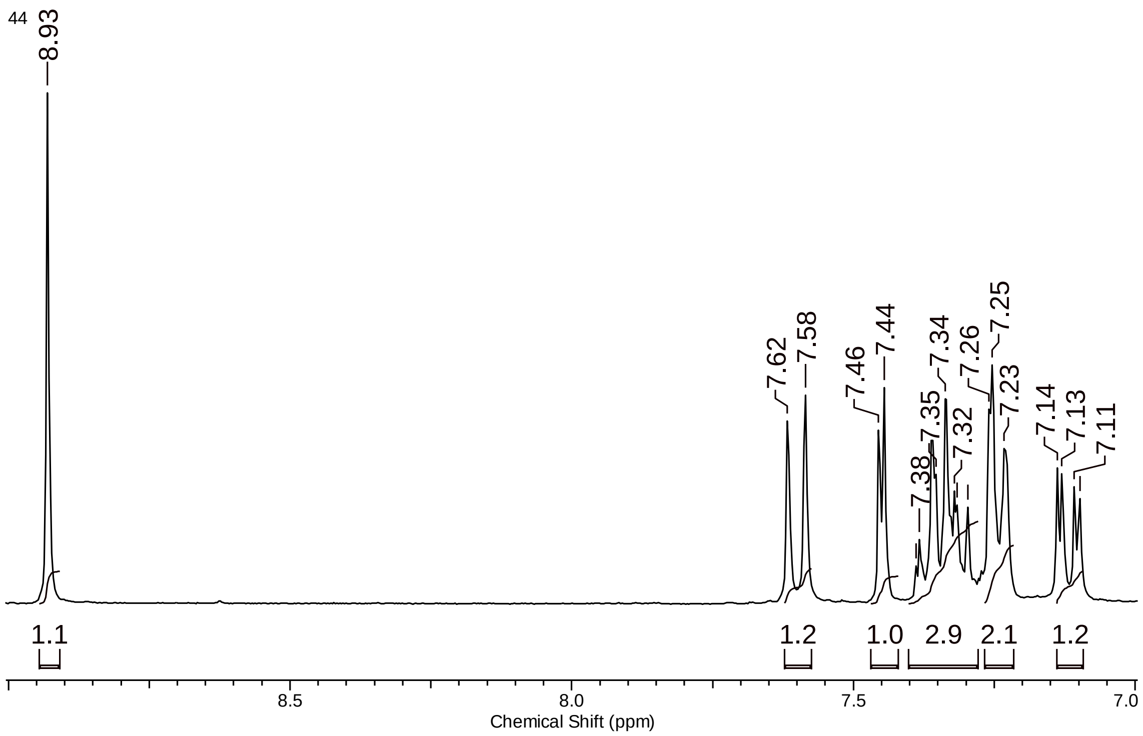

Figure S14. Expansion of the ^{1}H NMR spectrum of derivative **6d** (DMSO-d_6, 300.00 MHz).

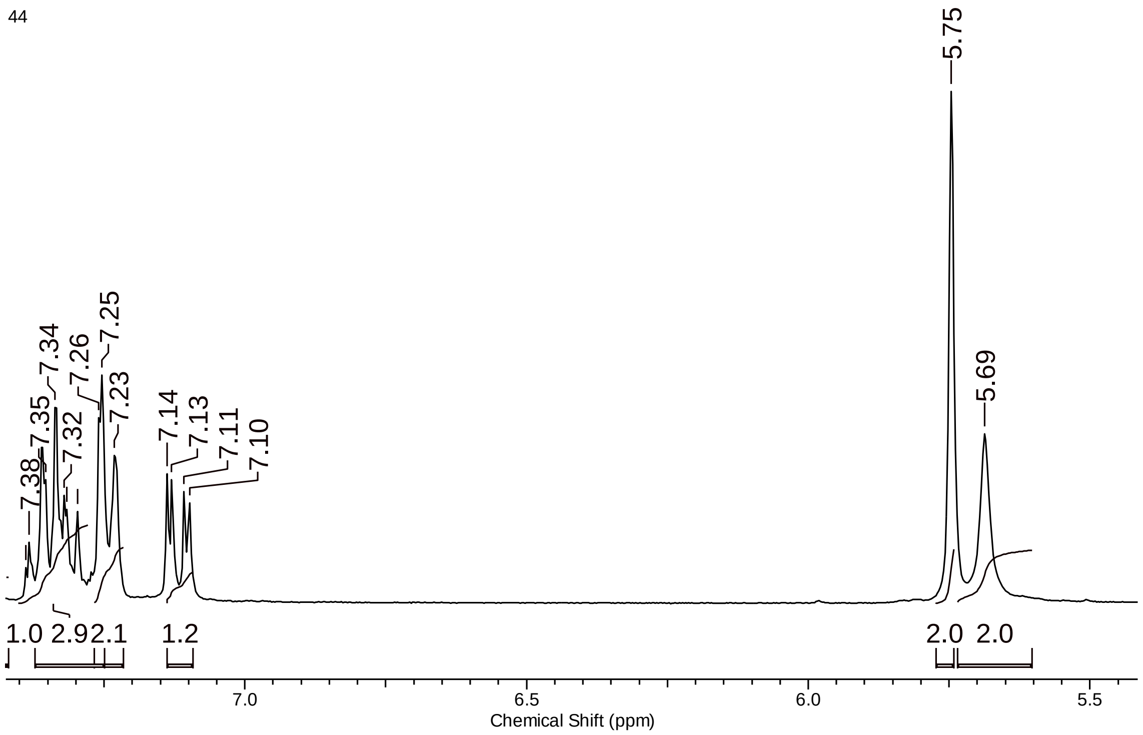

Figure S15. Expansion of the ^{1}H NMR spectrum of derivative **6d** (DMSO-d_6, 300.00 MHz).

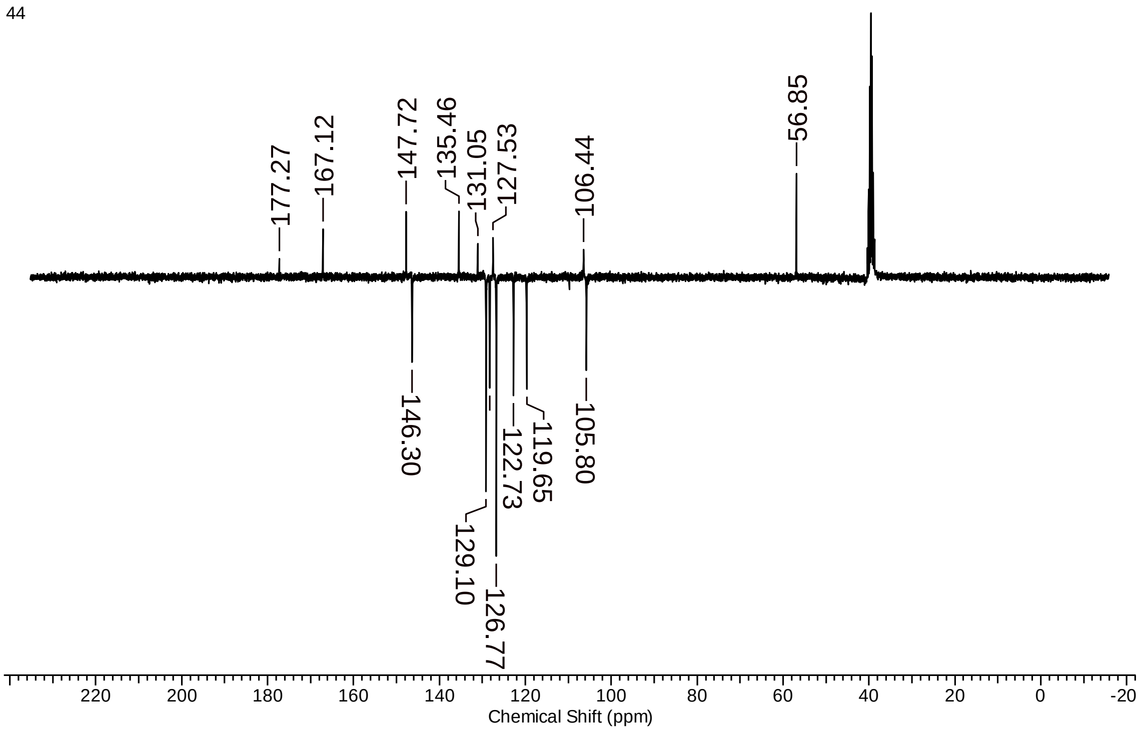

Figure S16. ^{13}C-APT NMR spectrum of derivative **6d** (DMSO-d_6, 75.00 MHz).

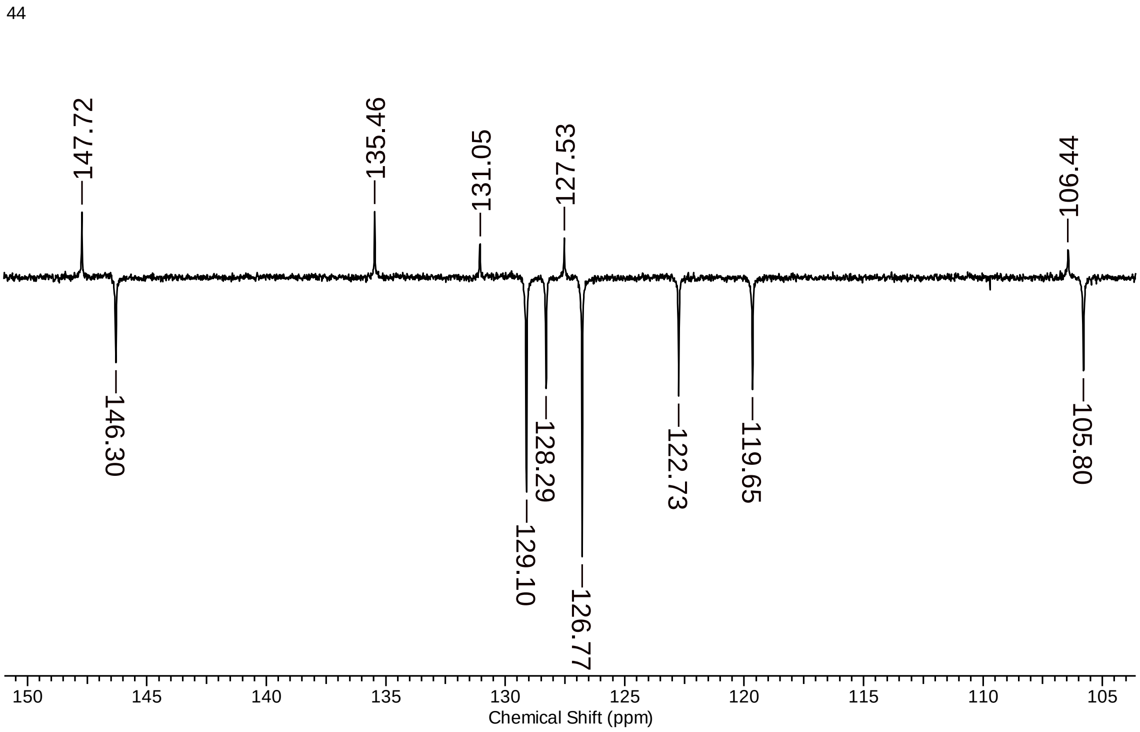

Figure S17. Expansion of the ^{13}C-APT NMR spectrum of derivative **6d** (DMSO-d_6, 75.00 MHz).

I.D) 2,5-dichloro-3,6-bis((3-(ethoxycarbonyl)-1-ethyl-4-oxo-1,4-dihydroquinolin-6-yl)amino)-1,4-benzoquinone (8a)

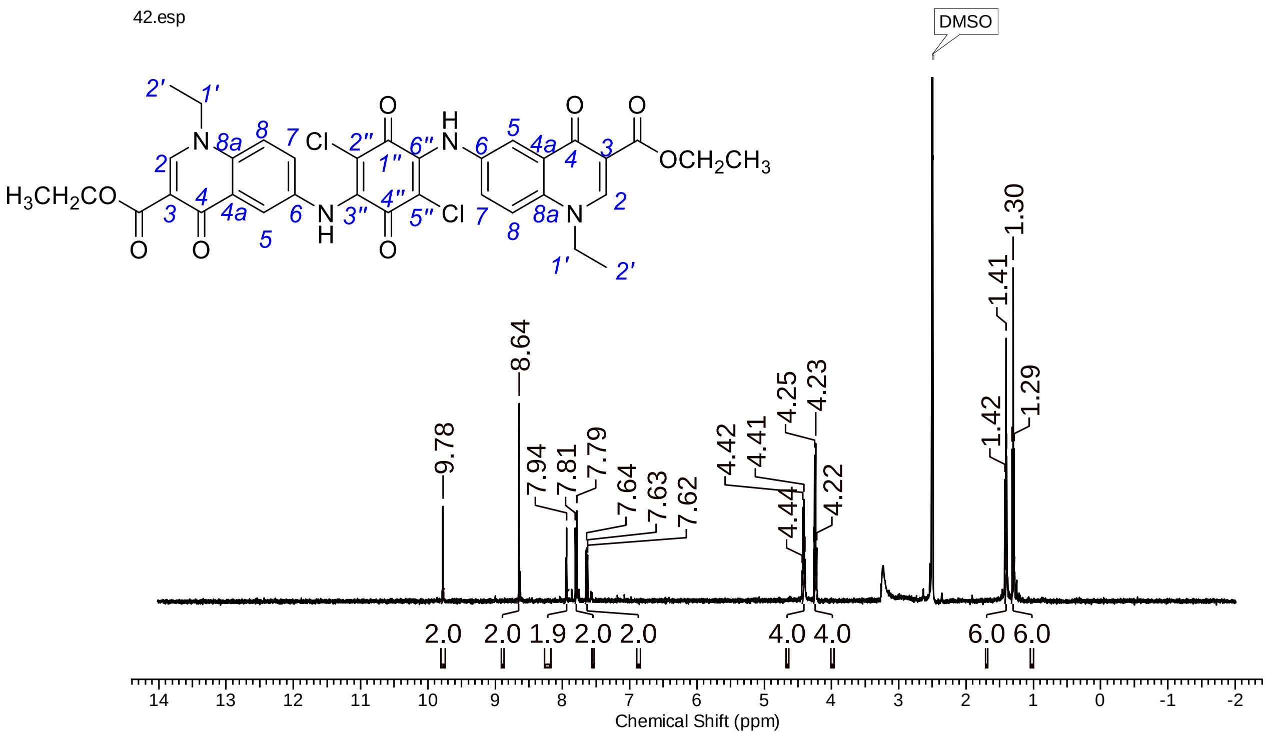

Figure S18. ^{1}H NMR spectrum of derivative **8a** (DMSO-*d*$_6$, 500.00 MHz).

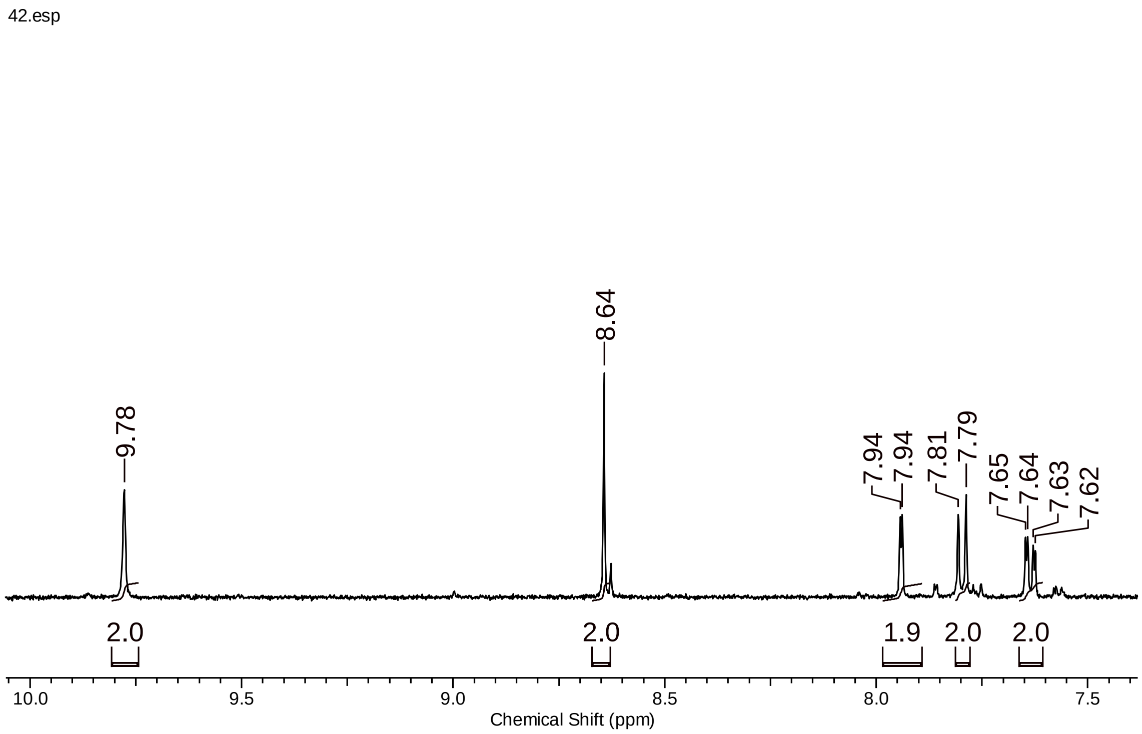

Figure S19. Expansion of the ^{1}H NMR spectrum of derivative **8a** (DMSO-*d*$_6$, 500.00 MHz).

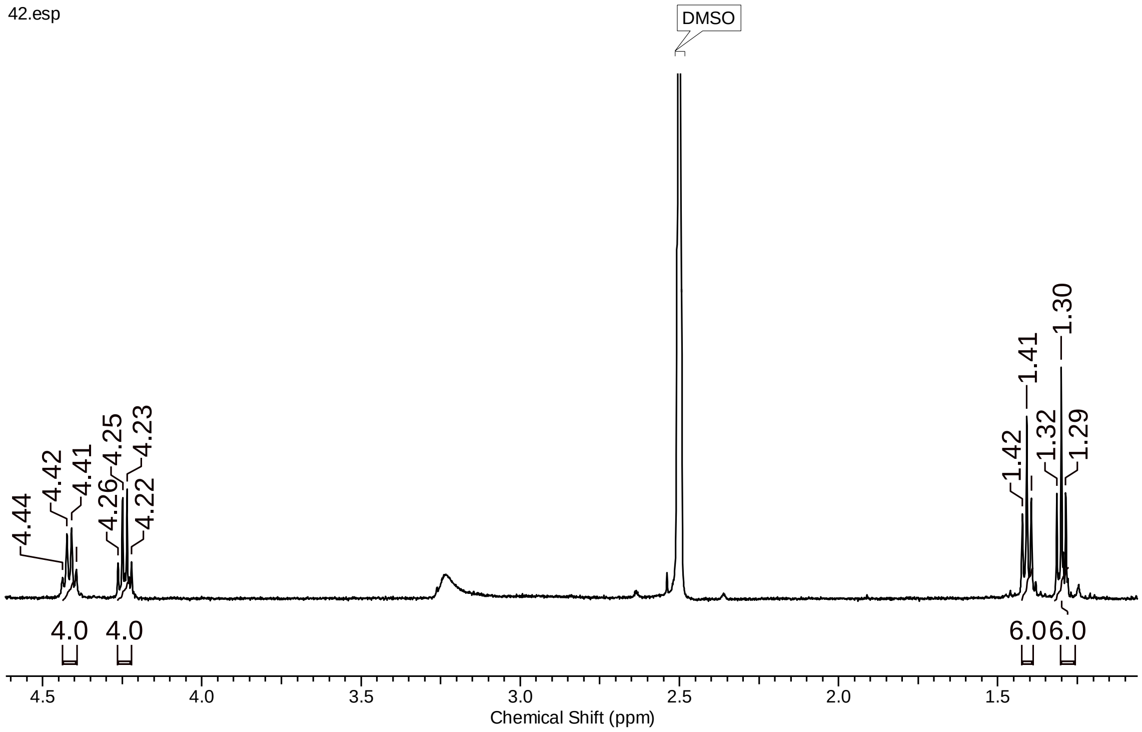

Figure S20. Expansion of the ^{1}H NMR spectrum of derivative **8a** (DMSO-d_6, 500.00 MHz).

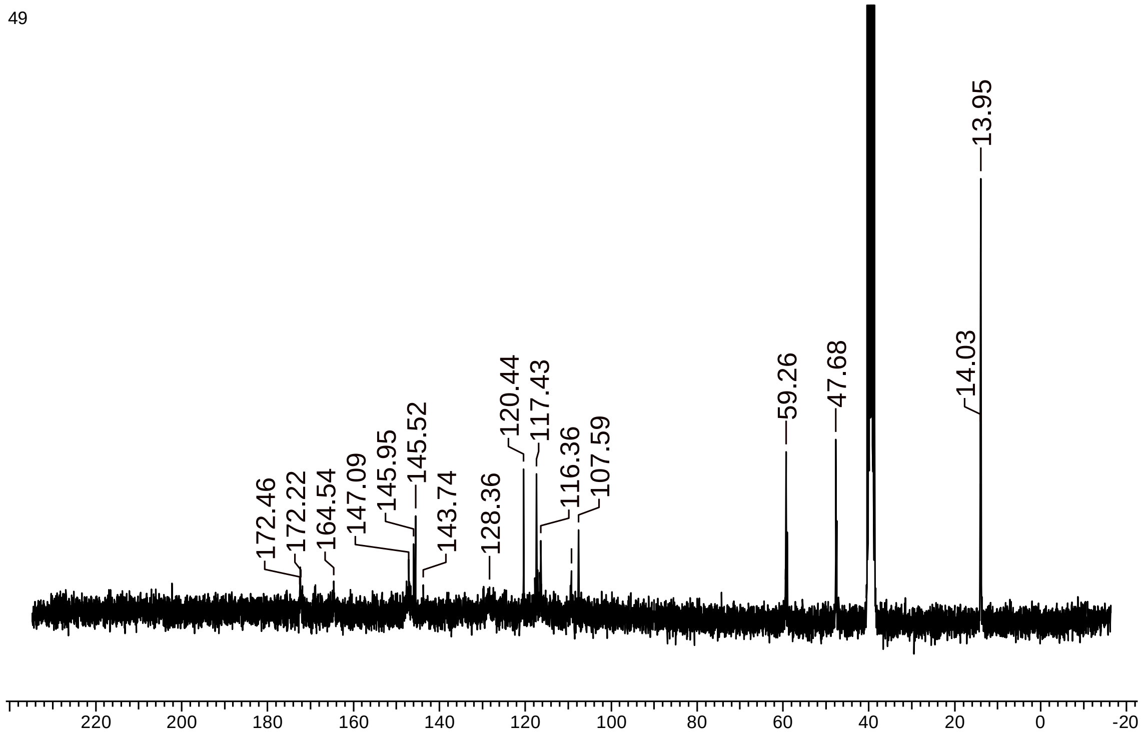

Figure S21. ^{13}C NMR spectrum of derivative **8a** (DMSO-d_6, 4 eq. K_2CO_3, 75.00 MHz).

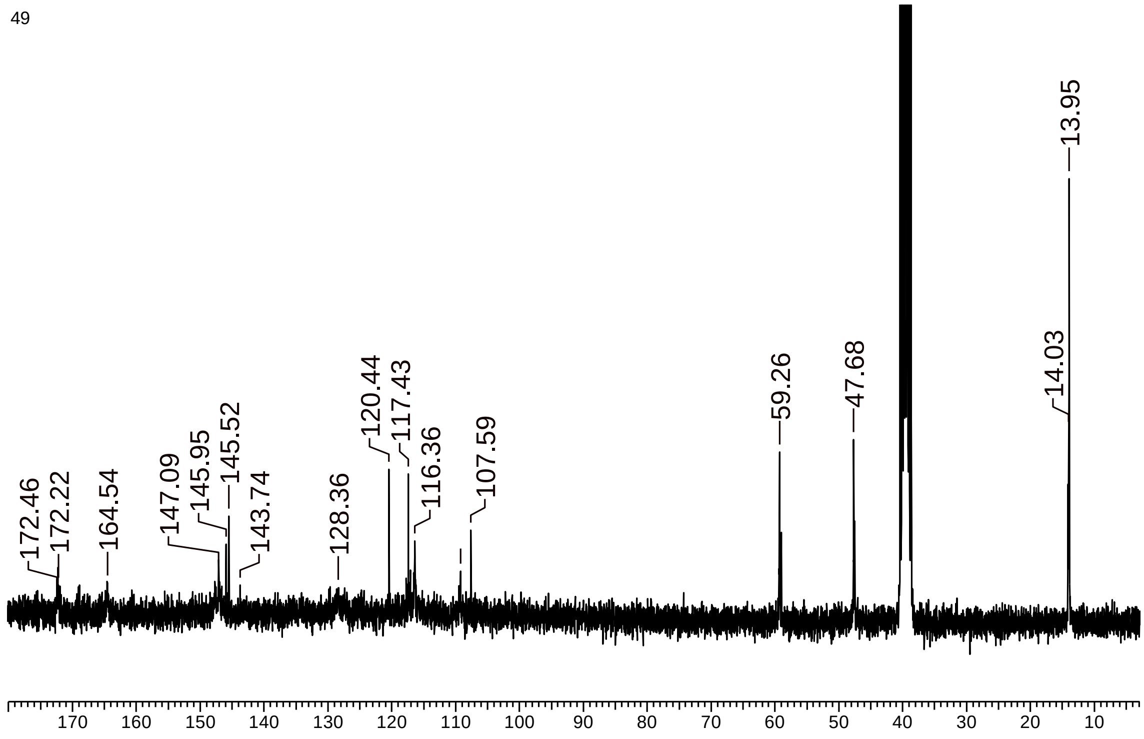

Figure S22. Expansion of the ^{13}C NMR spectrum of derivative **8a** (DMSO-d_6, 4 eq. K_2CO_3, 75.00 MHz).

I.E) 2,5-dichloro-3,6-bis((3-(ethoxycarbonyl)-4-oxo-1-propyl-1,4-dihydroquinolin-6-yl)amino)-1,4-benzoquinone (8b)

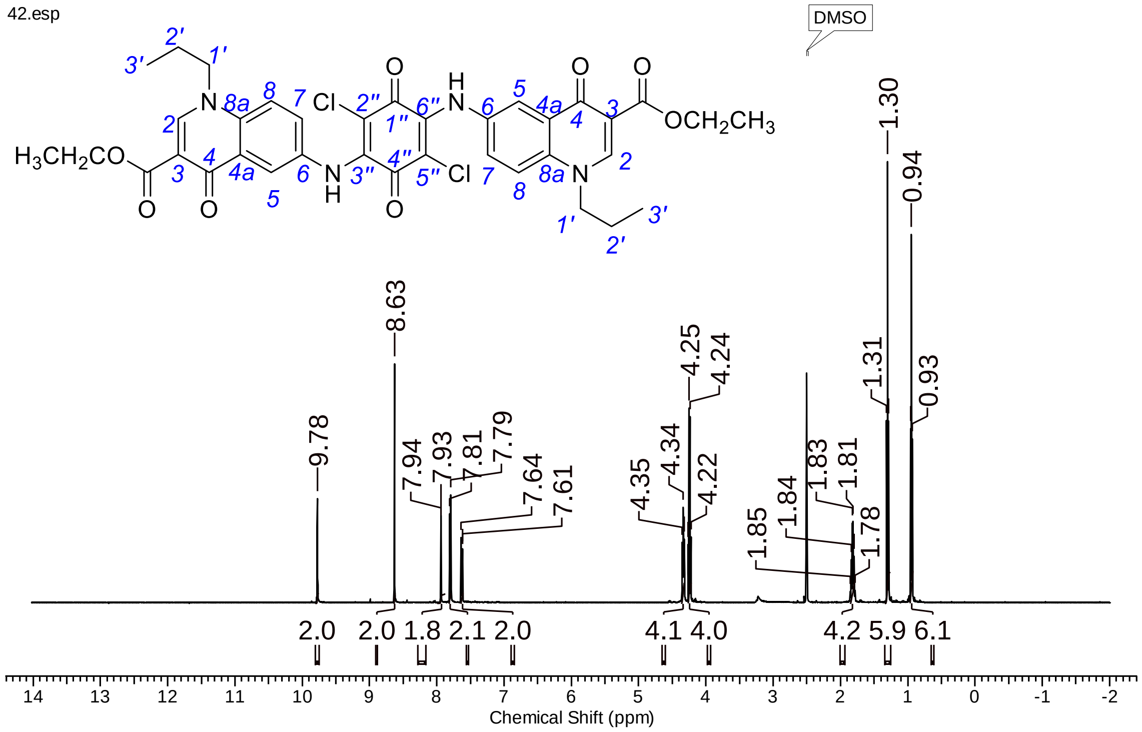

Figure S23. ^{1}H NMR spectrum of derivative **8b** (DMSO-*d*$_6$, 500.00 MHz).

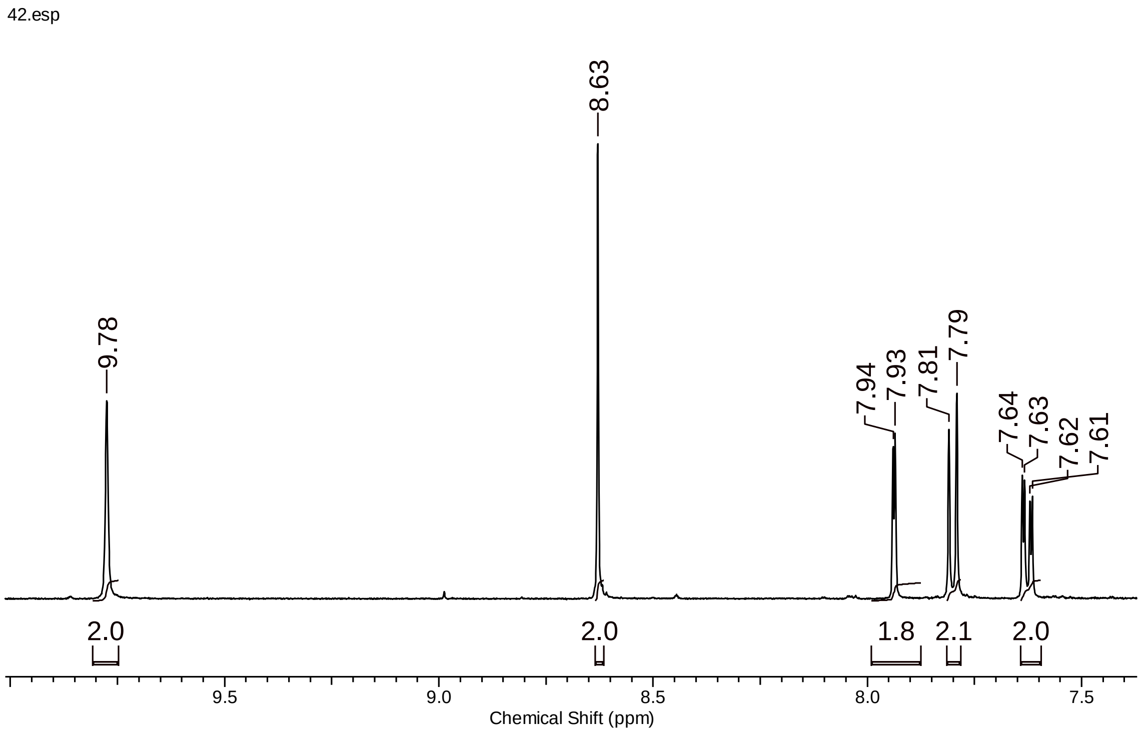

Figure S24. Expansion of the ^{1}H NMR spectrum of derivative **8b** (DMSO-*d*$_6$, 500.00 MHz).

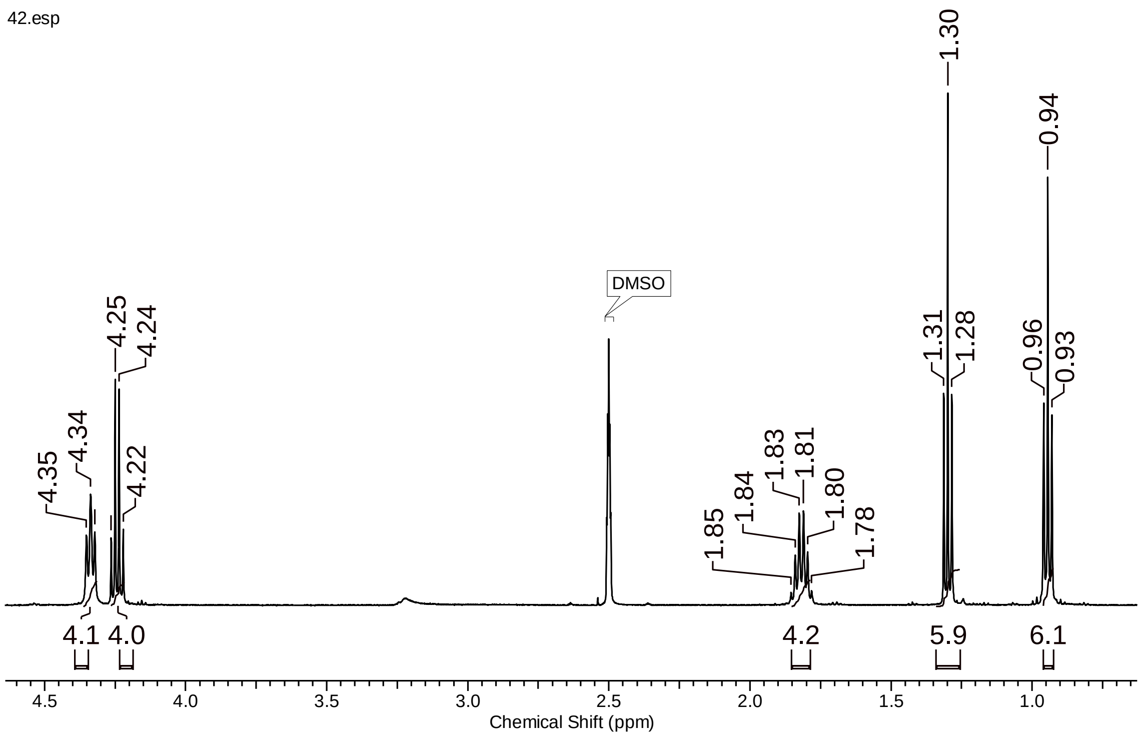

Figure S25. Expansion of the ^{1}H NMR spectrum of derivative **8b** (DMSO-*d*$_6$, 500.00 MHz).

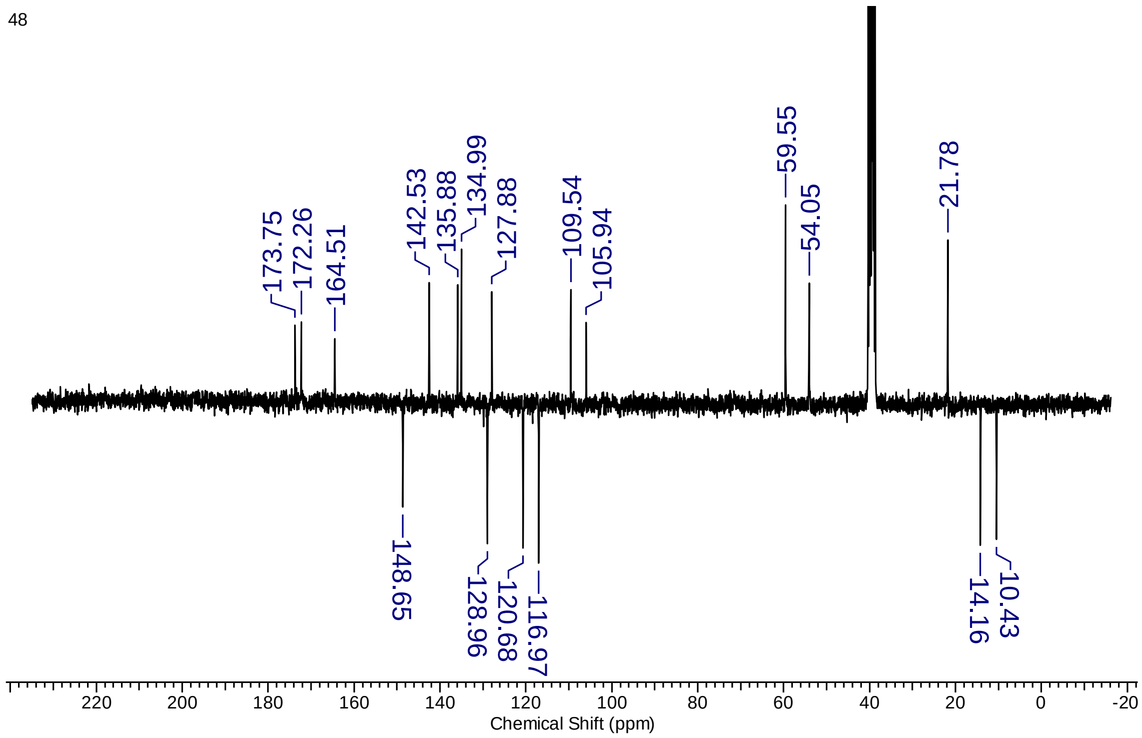

Figure S26. ^{13}C-APT NMR spectrum of derivative **8b** (DMSO-d_6, 75.00 MHz).

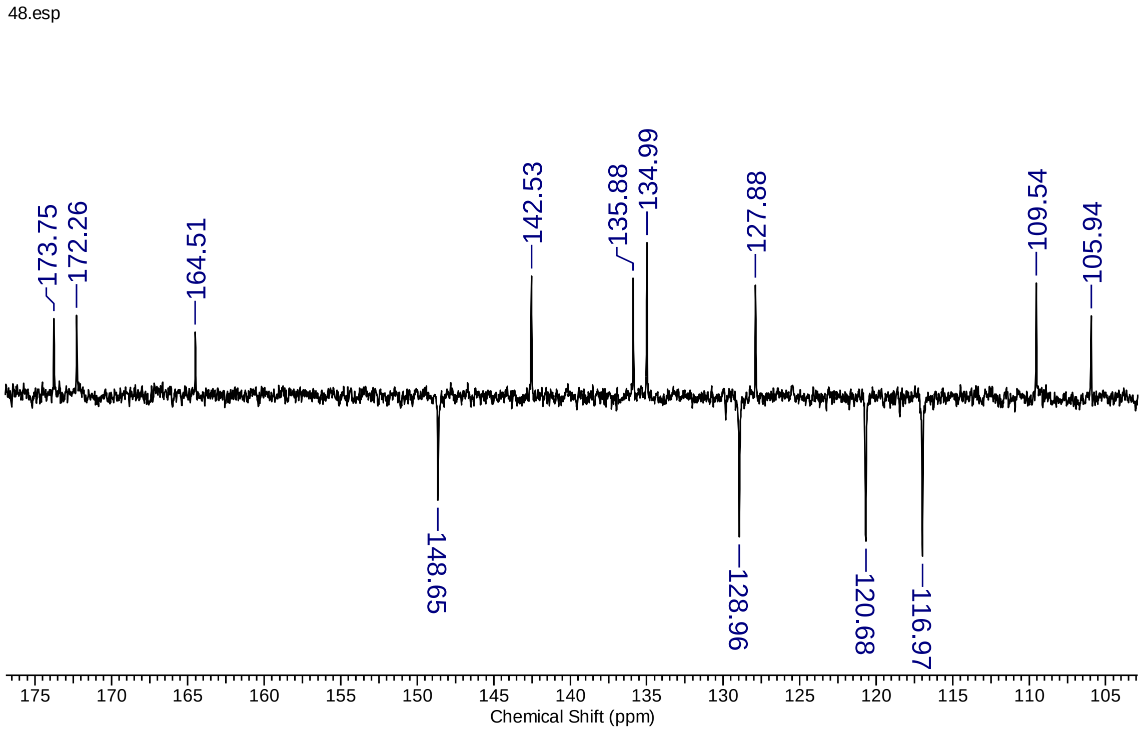

Figure S27. Expansion of the ^{13}C-APT NMR spectrum of derivative **8b** (DMSO-d_6, 75.00 MHz).

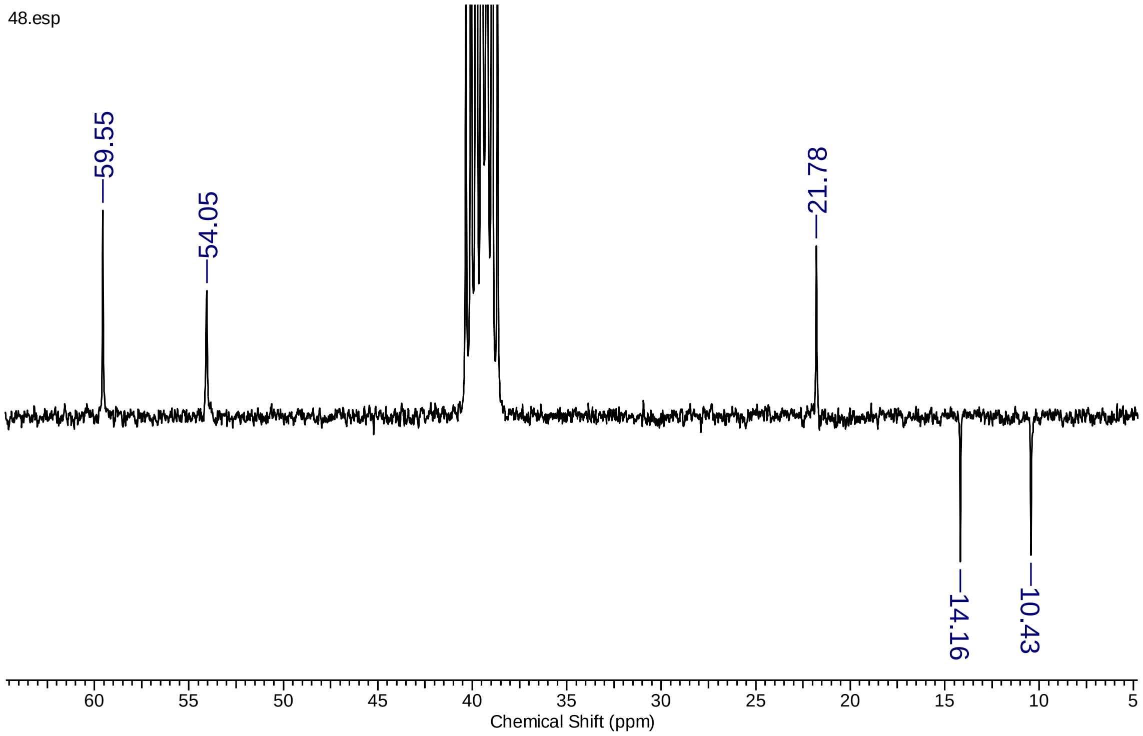

Figure S28. Expansion of the ^{13}C-APT NMR spectrum of derivative **8b** (DMSO-d_6, 75.00 MHz).

I.F) 2,5-dichloro-3,6-bis((3-(ethoxycarbonyl)-4-oxo-1-pentyl-1,4-dihydroquinolin-6-yl)amino)-1,4-benzoquinone (8c)

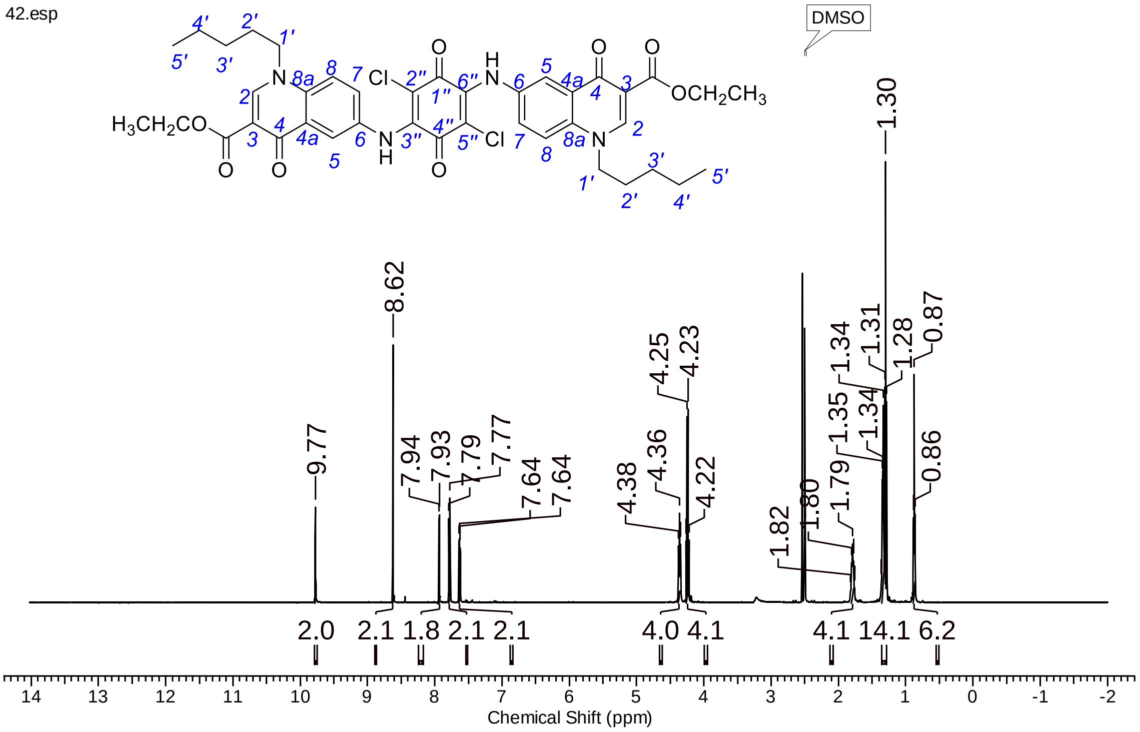

Figure S29. ^{1}H NMR spectrum of derivative **8c** (DMSO-d_6, 500.00 MHz).

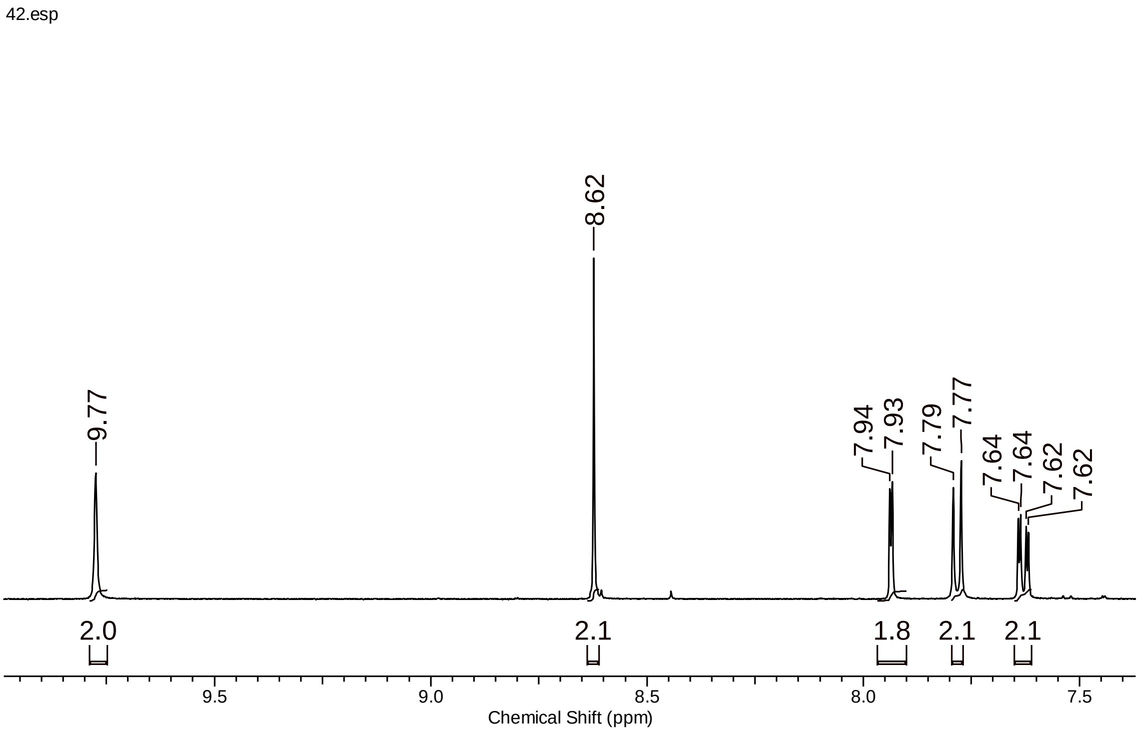

Figure S30. Expansion of the ^{1}H NMR spectrum of derivative **8c** (DMSO-d_6, 500.00 MHz).

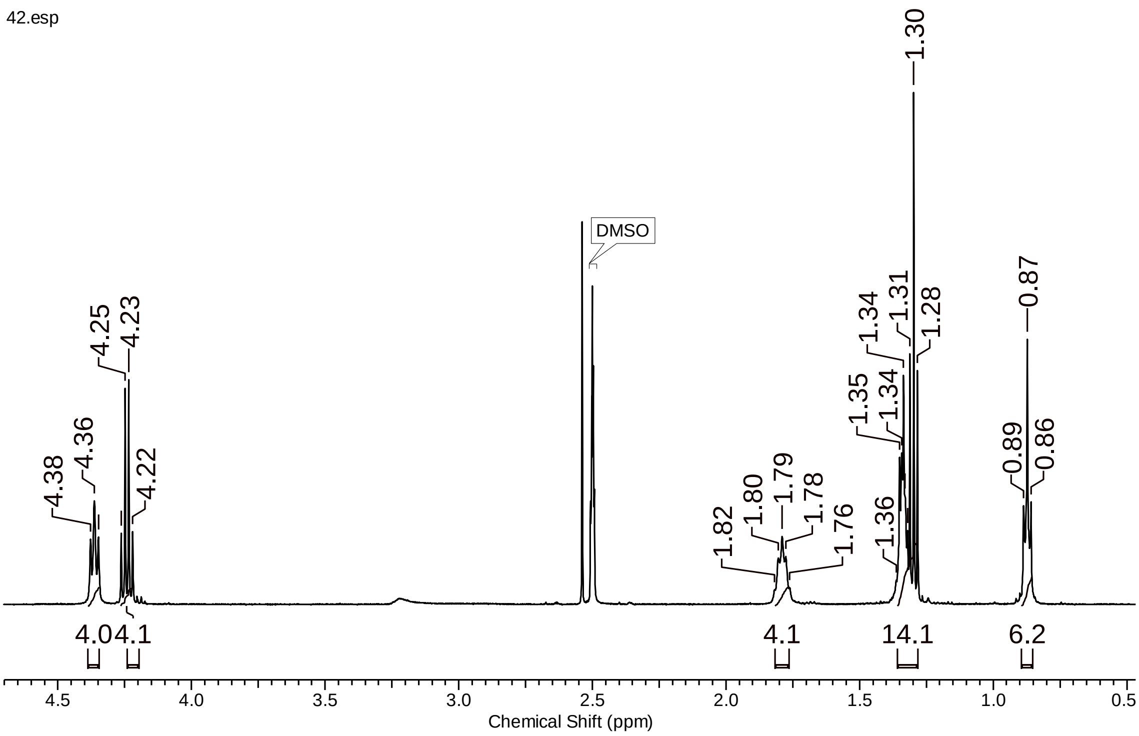

Figure S31. Expansion of the ^{1}H NMR spectrum of derivative **8c** (DMSO-d_6, 500.00 MHz).

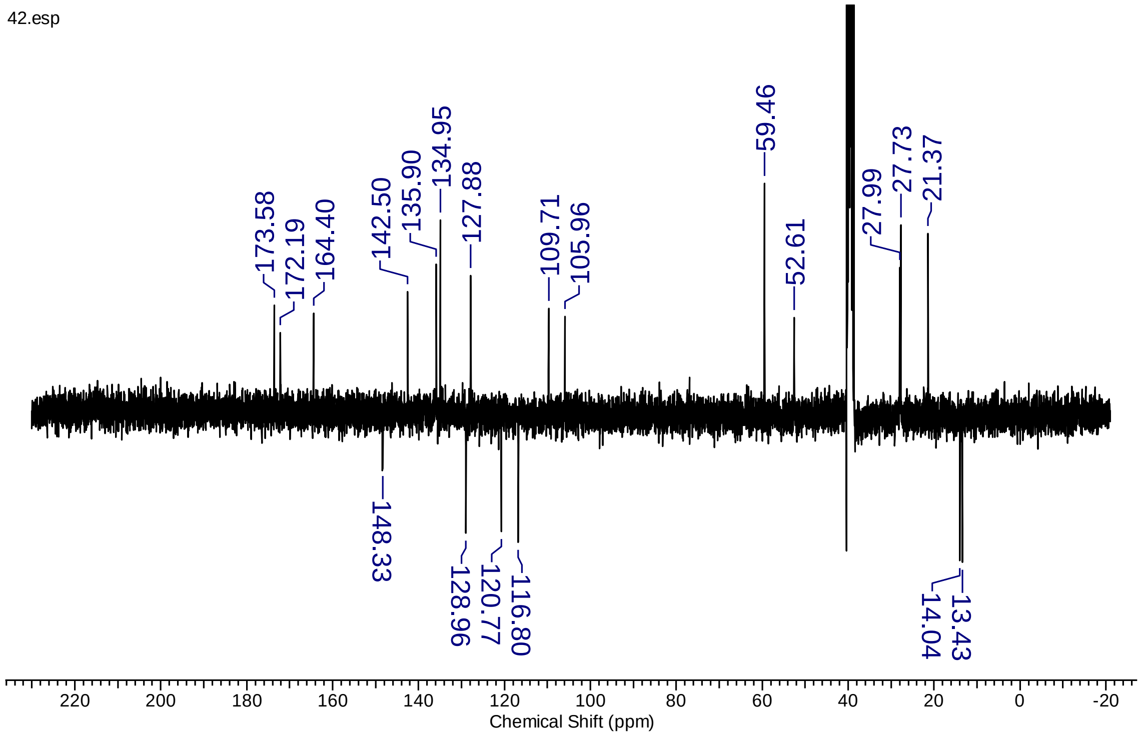

Figure S32. ^{13}C-APT NMR spectrum of derivative **8c** (DMSO-d_6, 75.00 MHz).

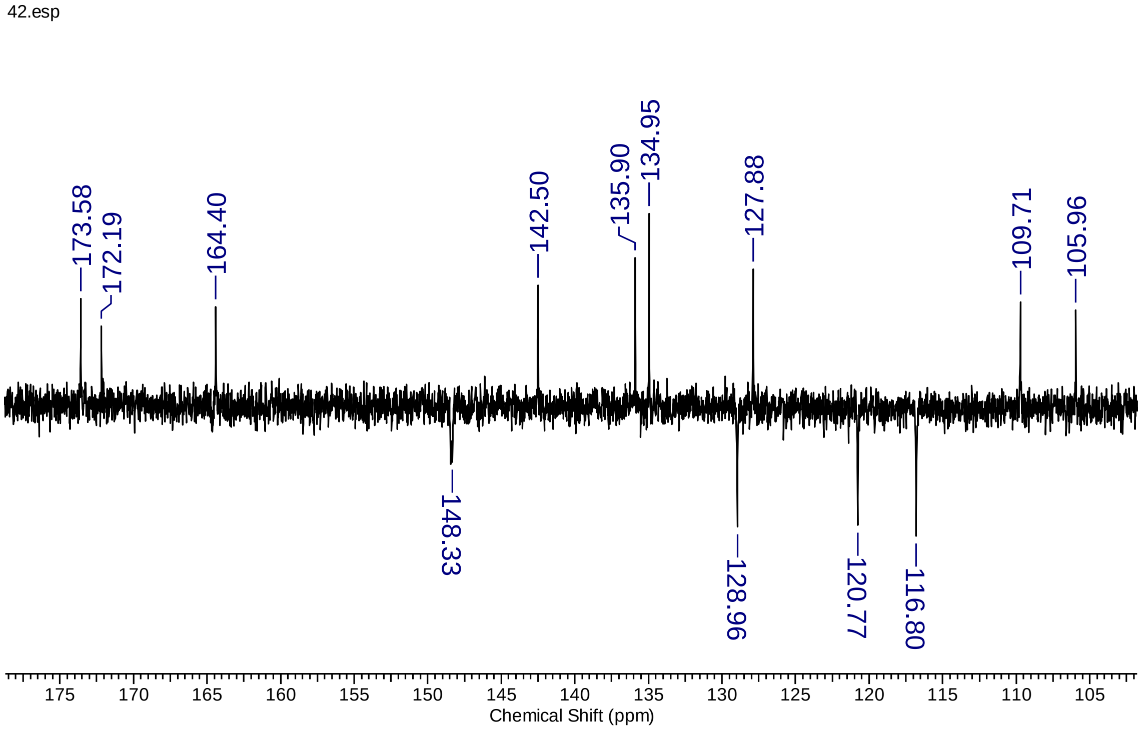

Figure S33. Expansion of the ^{13}C-APT NMR spectrum of derivative **8c** (DMSO-d_6, 75.00 MHz).

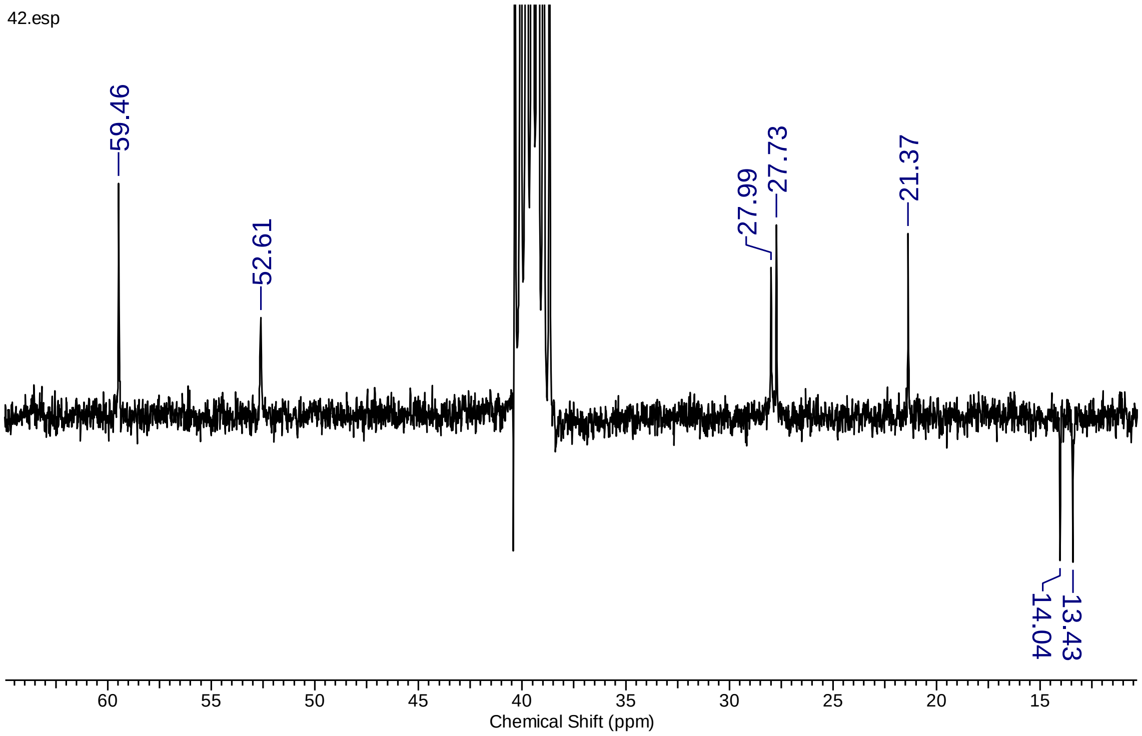

Figure S34. Expansion of the ^{13}C-APT NMR spectrum of derivative **8c** (DMSO-d_6, 75.00 MHz).

I.G) 2,5-dichloro-3,6-bis((3-carboxy-4-oxo-1-propyl-1,4-dihydroquinolin-6-yl)amino)-1,4-benzoquinone (8d)

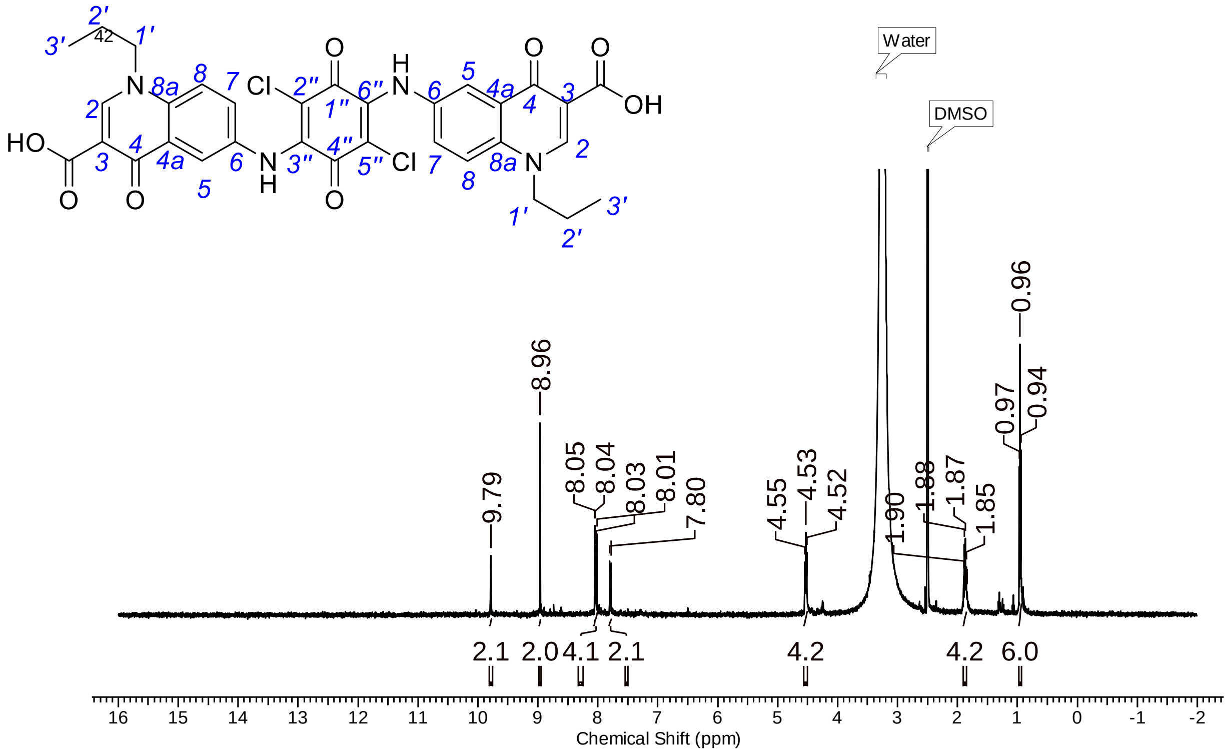

Figure S35. ^{1}H NMR spectrum of derivative **8d** (DMSO-d_6, 500.00 MHz).

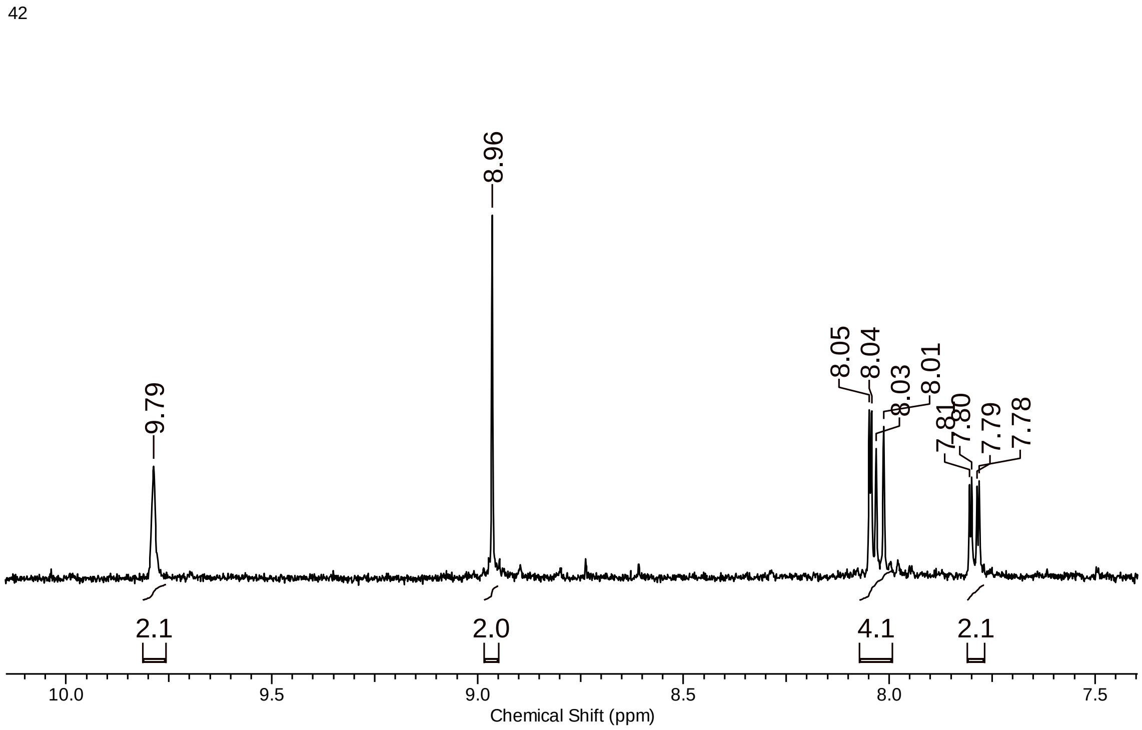

Figure S36. Expansion of the ^{1}H NMR spectrum of derivative **8d** (DMSO-d_6, 500.00 MHz).

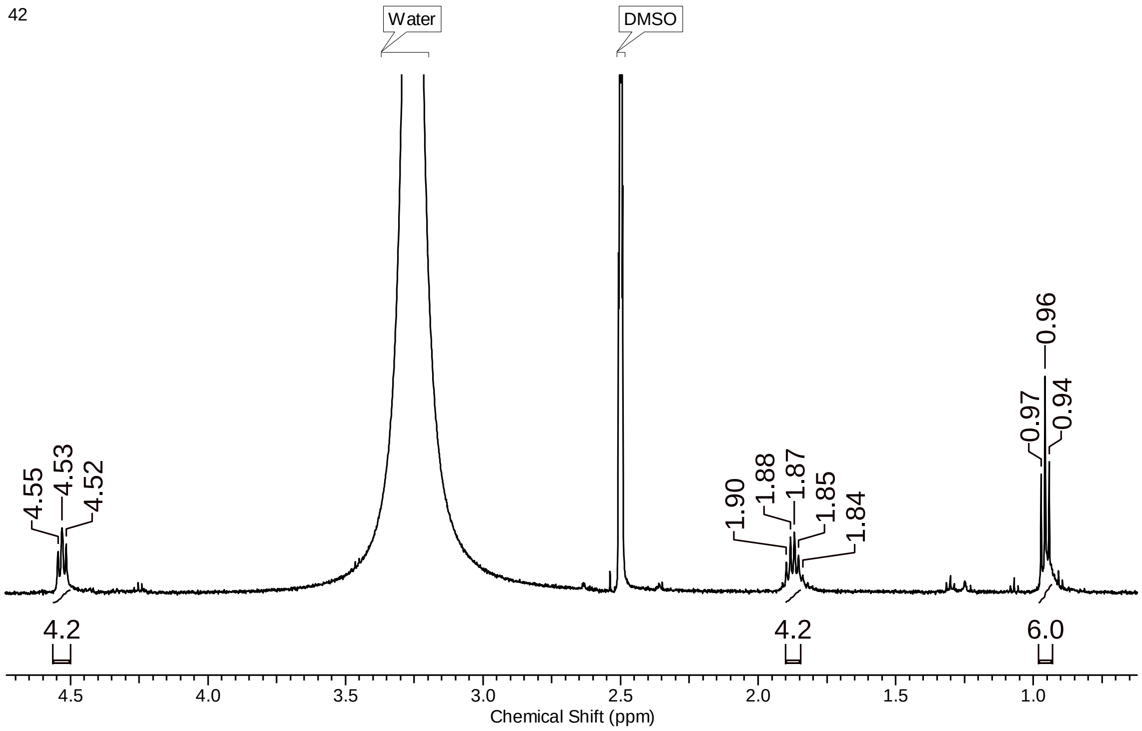

Figure S37. Expansion of the ^{1}H NMR spectrum of derivative **8d** (DMSO-d_6, 500.00 MHz).

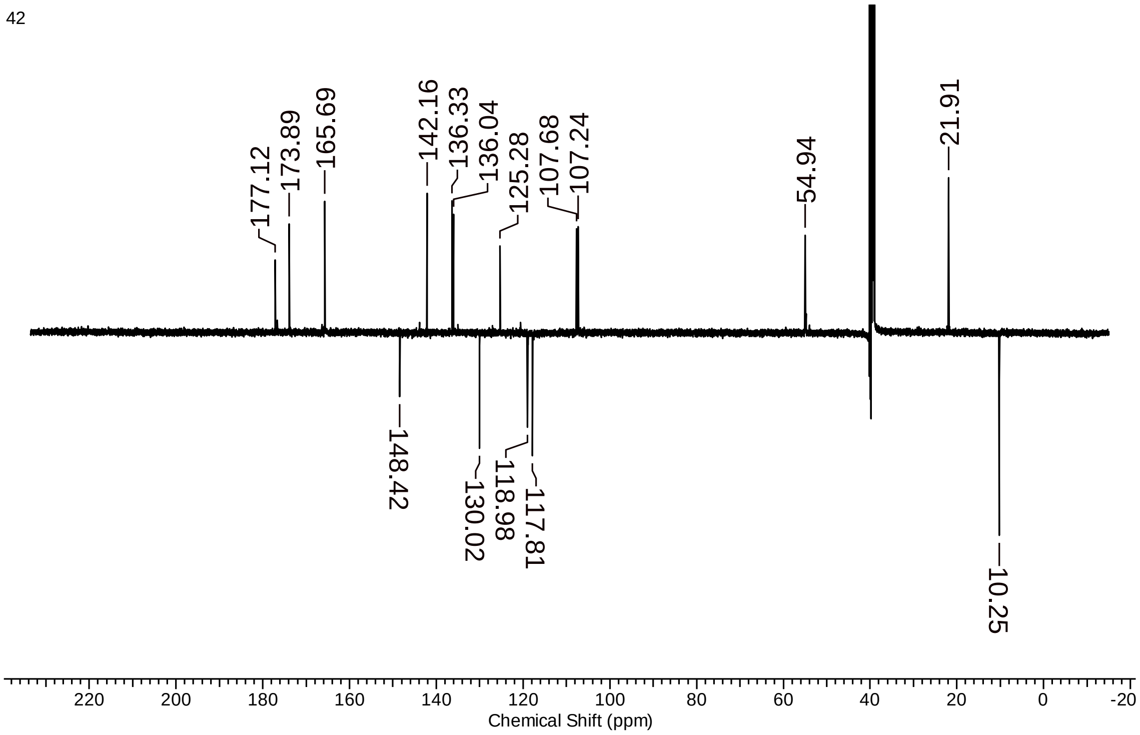

Figure S38. ^{13}C-APT NMR spectrum of derivative **8d** (DMSO-d_6, 125.00 MHz).

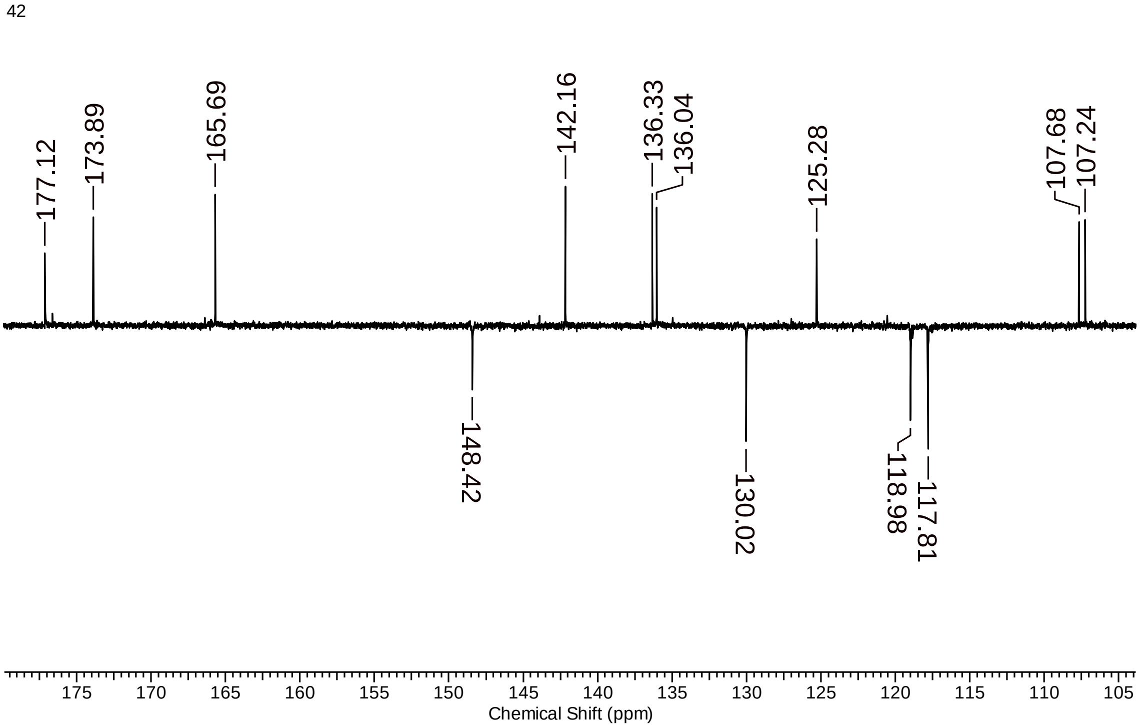

Figure S39. Expansion of the ^{13}C-APT NMR spectrum of derivative **8d** (DMSO-d_6, 125.00 MHz).

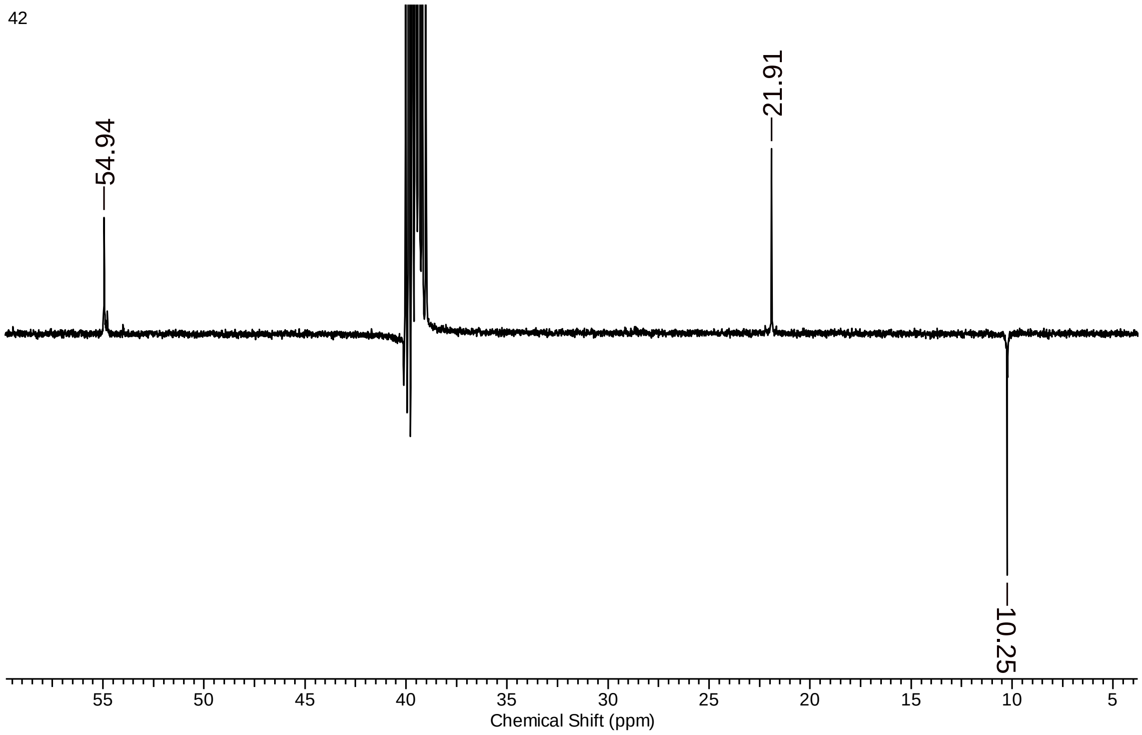

Figure S40. Expansion of the ^{13}C-APT NMR spectrum of derivative **8d** (DMSO-d_6, 125.00 MHz).

I.H) 2,5-dichloro-3,6-bis((3-carboxy-4-oxo-1-pentyl-1,4-dihydroquinolin-6-yl)amino)-1,4-benzoquinone (8e)

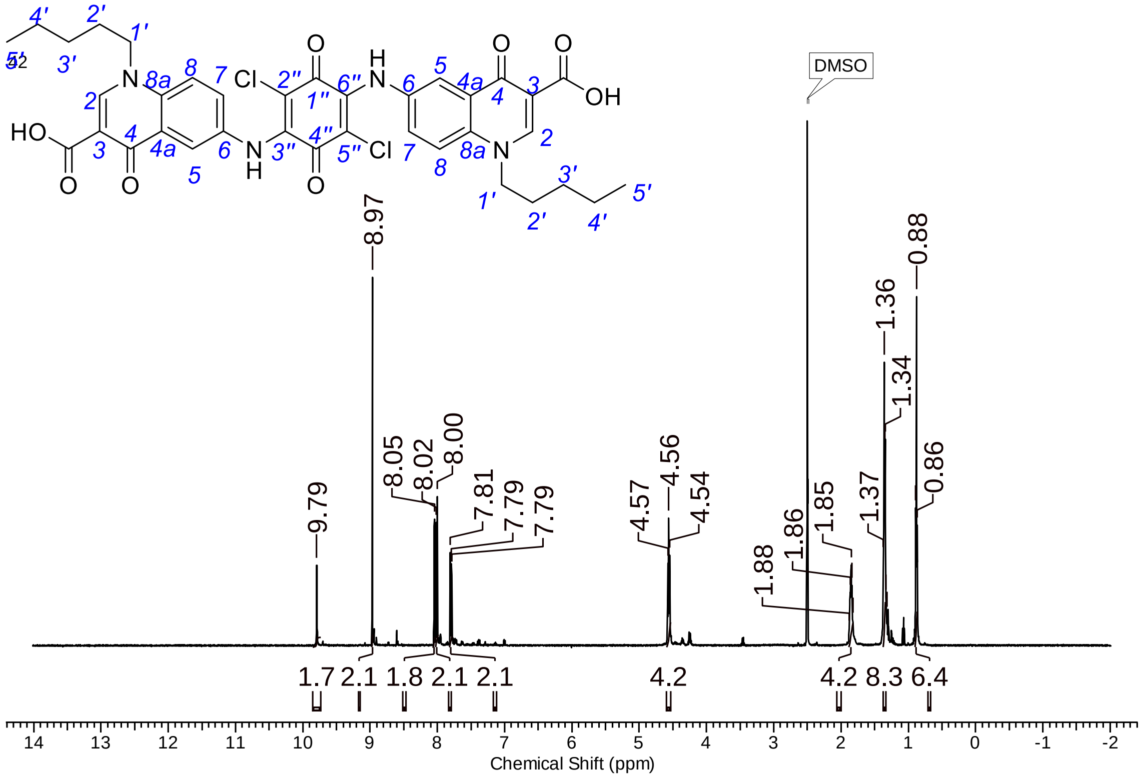

Figure S41. ^{1}H NMR spectrum of derivative **8e** (DMSO-d_6, 500.00 MHz).

42

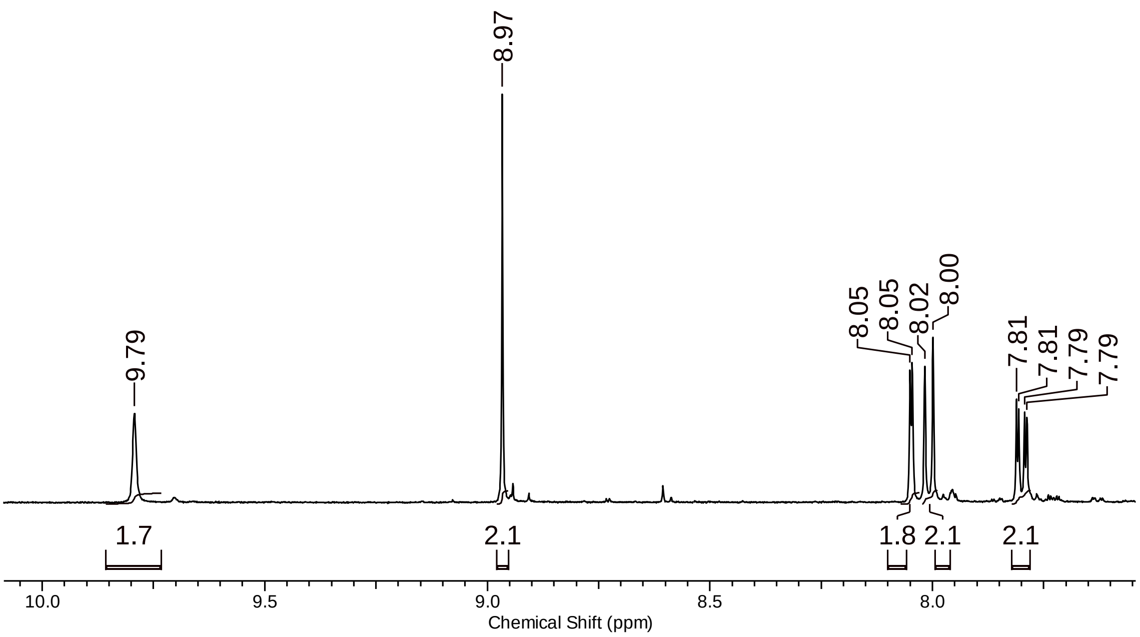

Figure S42. Expansion of the ^{1}H NMR spectrum of derivative **8e** (DMSO-*d*$_6$, 500.00 MHz).

42

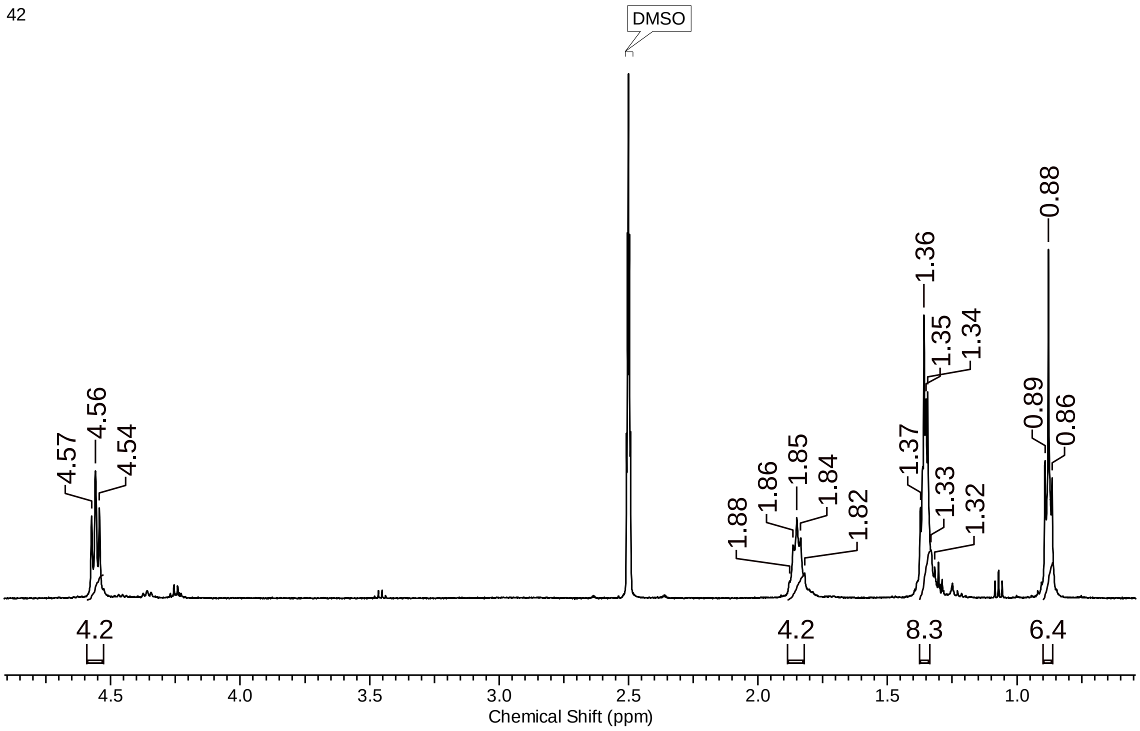

Figure S43. Expansion of the ^{1}H NMR spectrum of derivative **8e** (DMSO-*d*$_6$, 500.00 MHz).

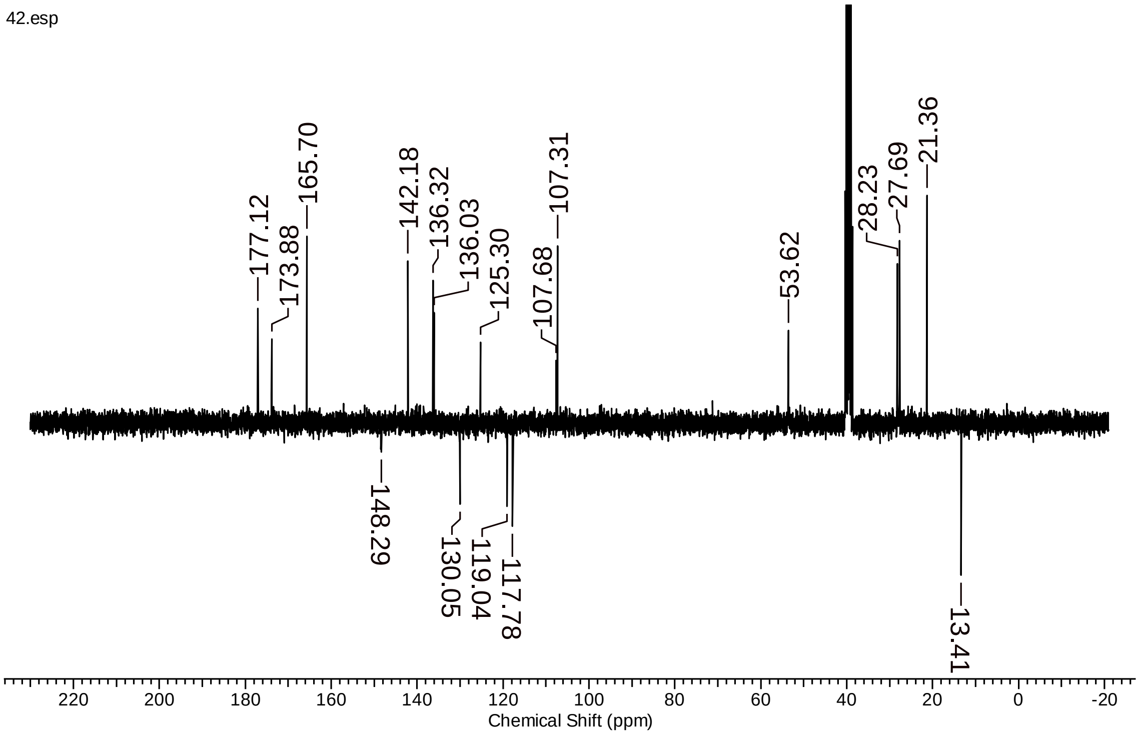

Figure S44. ^{13}C-APT NMR spectrum of derivative **8e** (DMSO-d_6, 75.00 MHz).

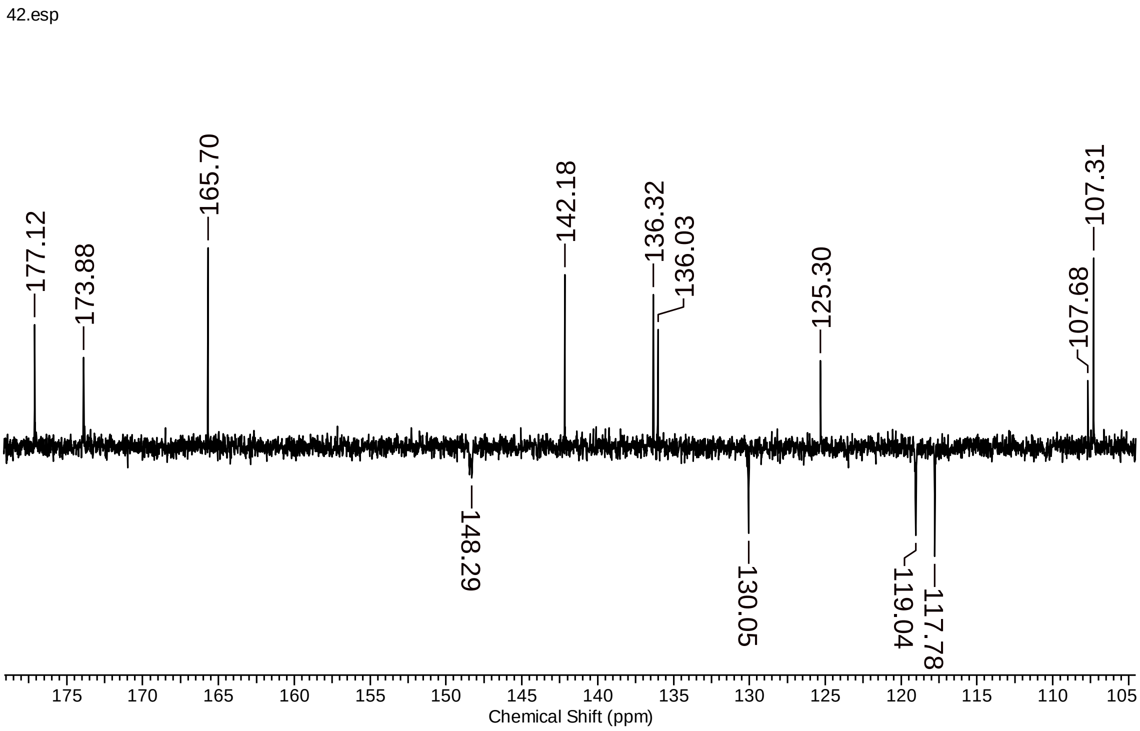

Figure S45. Expansion of the ^{13}C-APT NMR spectrum of derivative **8e** (DMSO-d_6, 75.00 MHz).

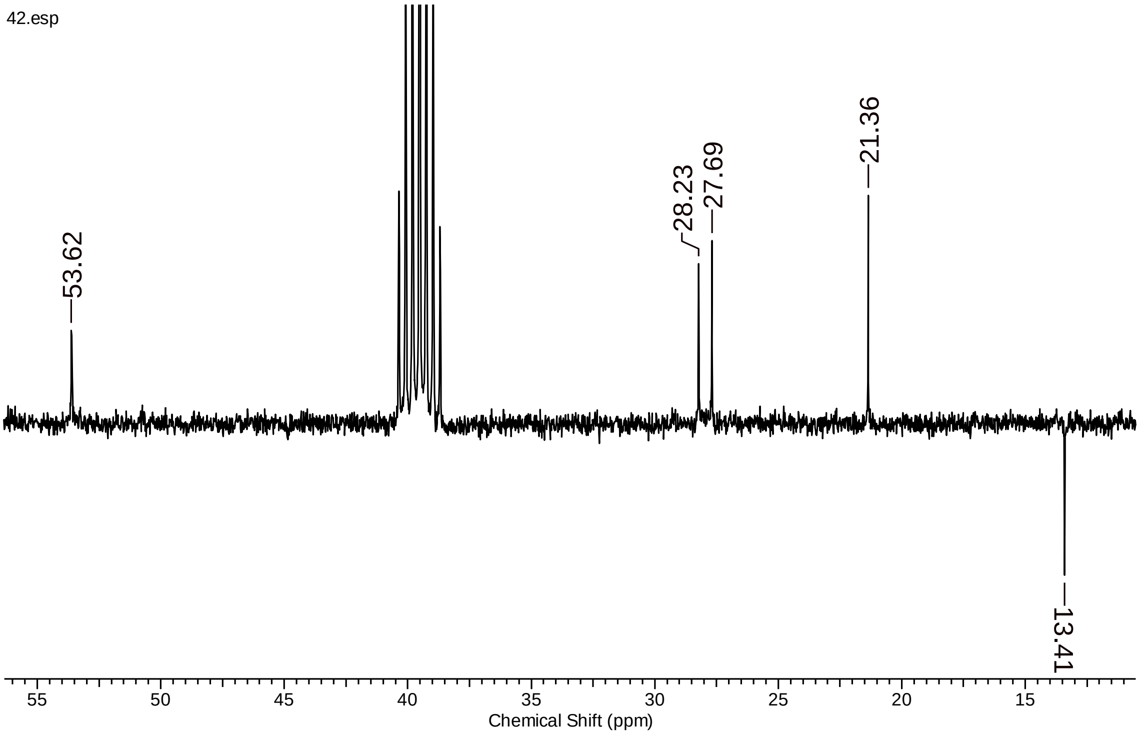

Figure S46. Expansion of the ^{13}C-APT NMR spectrum of derivative **8e** (DMSO-d_6, 75.00 MHz).

I.I) 2,5-dichloro-3,6-bis((1-benzyl-3-carboxy-4-oxo-1,4-dihydroquinolin-6-yl)amino)-1,4-benzoquinone (8f)

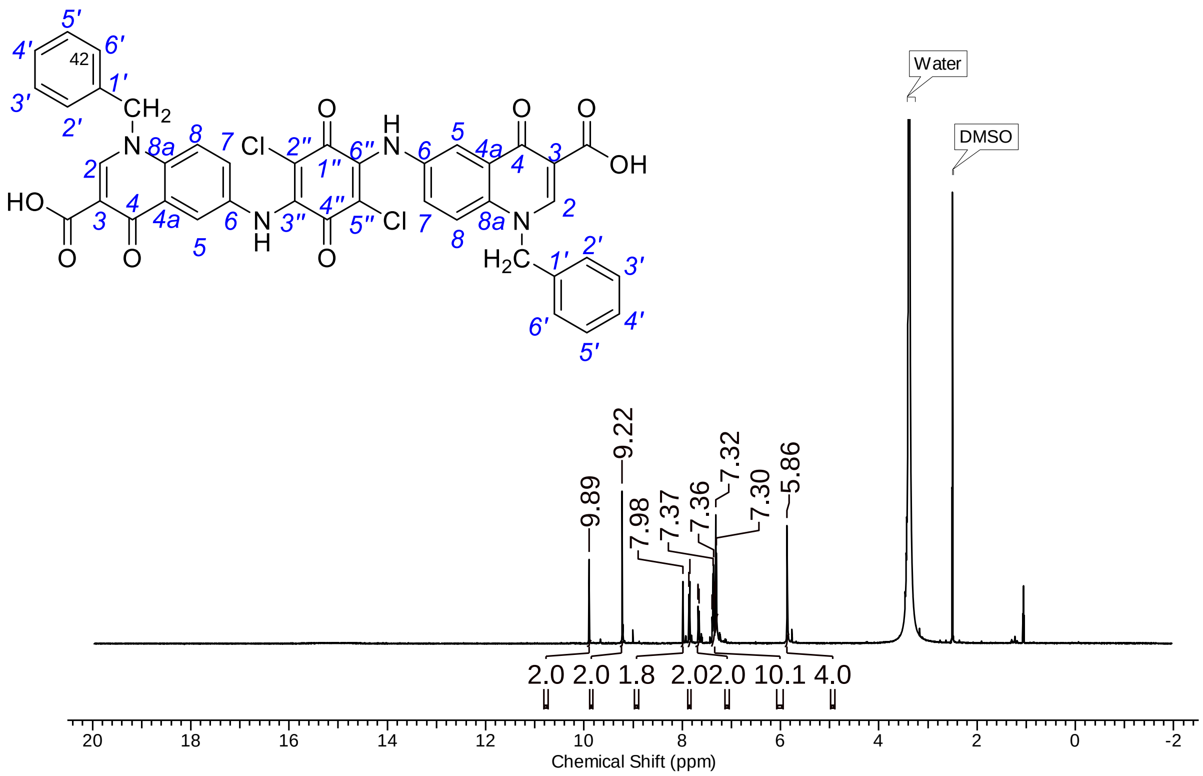

Figure S47. ^{1}H NMR spectrum of derivative **8f** (DMSO-d_6, 500.00 MHz).

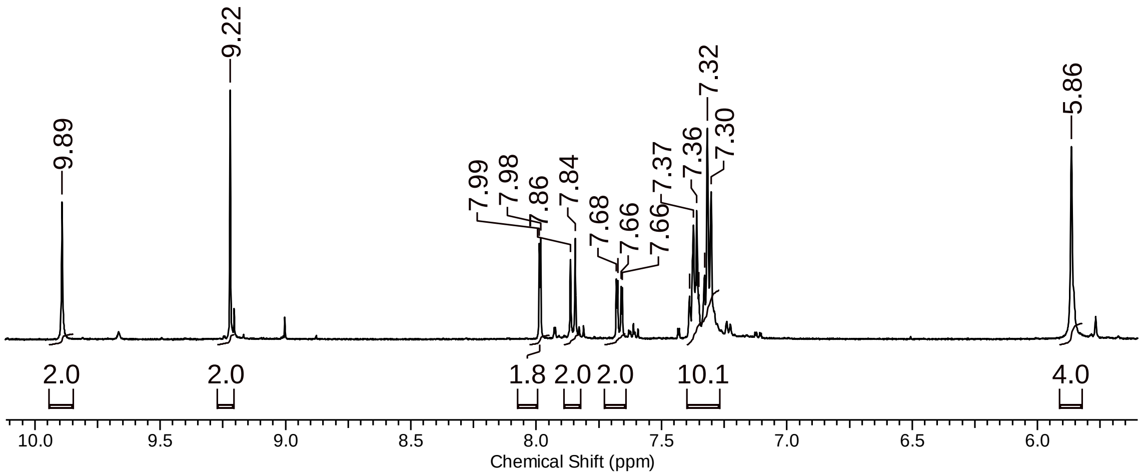

Figure S48. Expansion of the ^{1}H NMR spectrum of derivative **8f** (DMSO-d_6, 500.00 MHz).

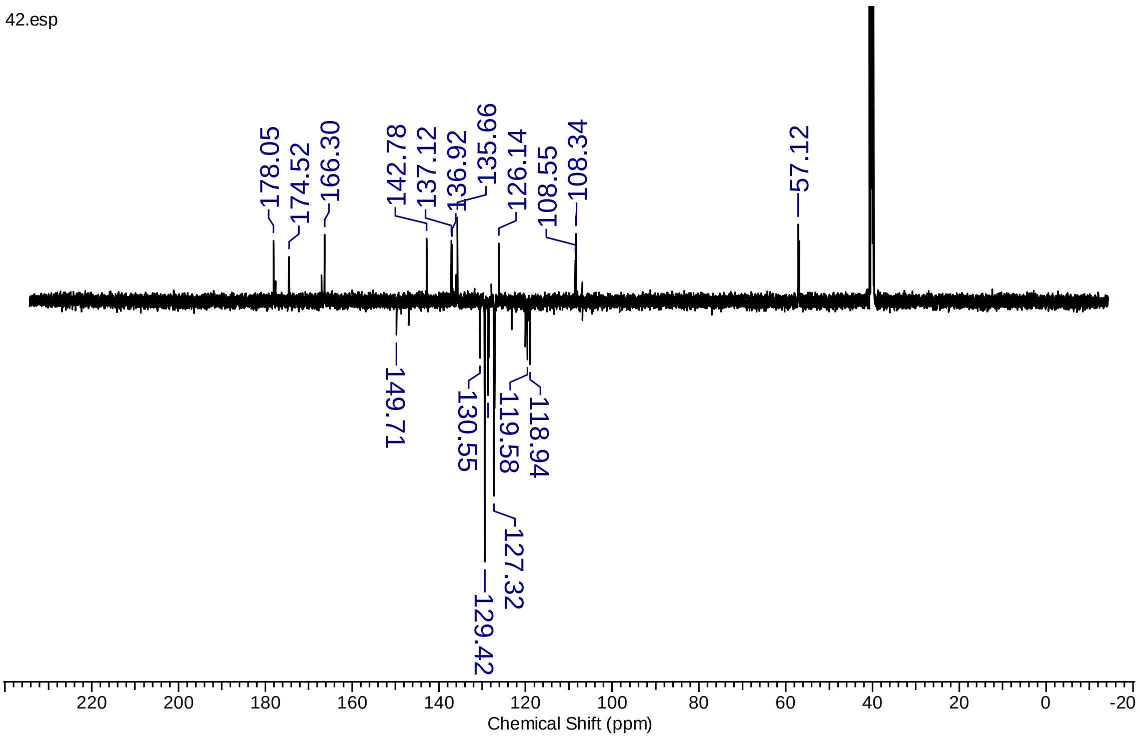

Figure S49. ^{13}C-APT NMR spectrum of derivative **8f** (DMSO-d_6, 125.00 MHz).

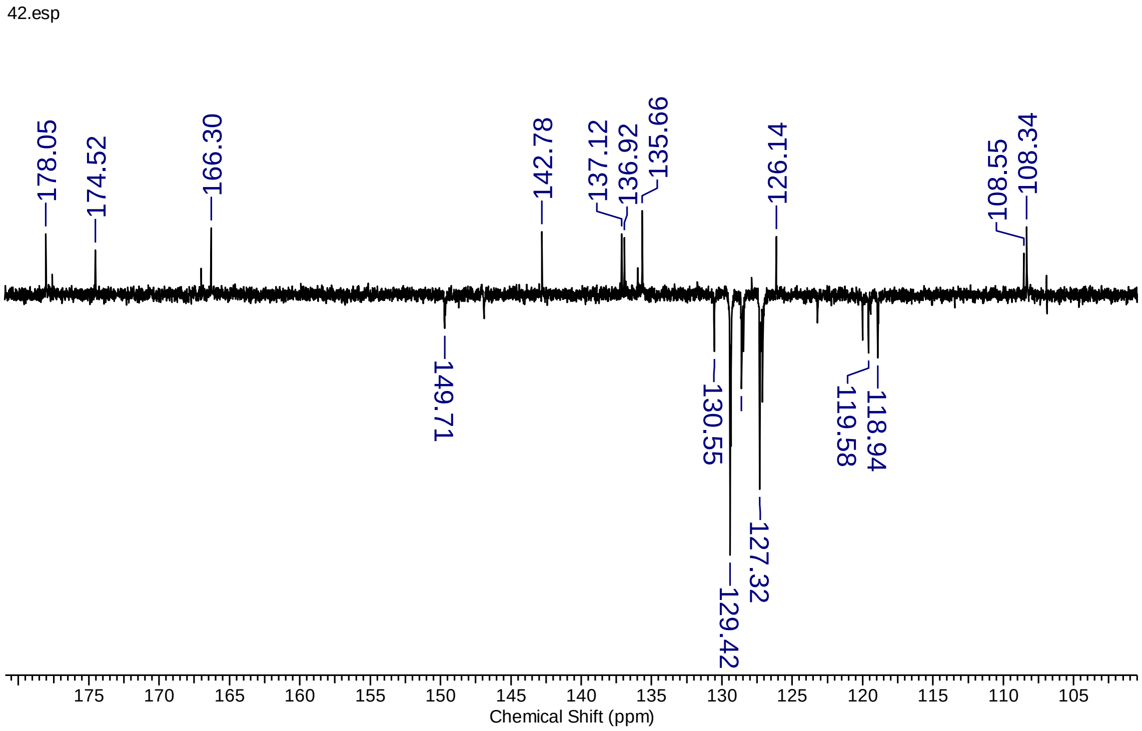

Figure S50. Expansion of the ^{13}C-APT NMR spectrum of derivative **8f** (DMSO-d_6, 125.00 MHz).

II) N2a cells cell viability assay in the presence of aggregates produced in RT-QuIC assays

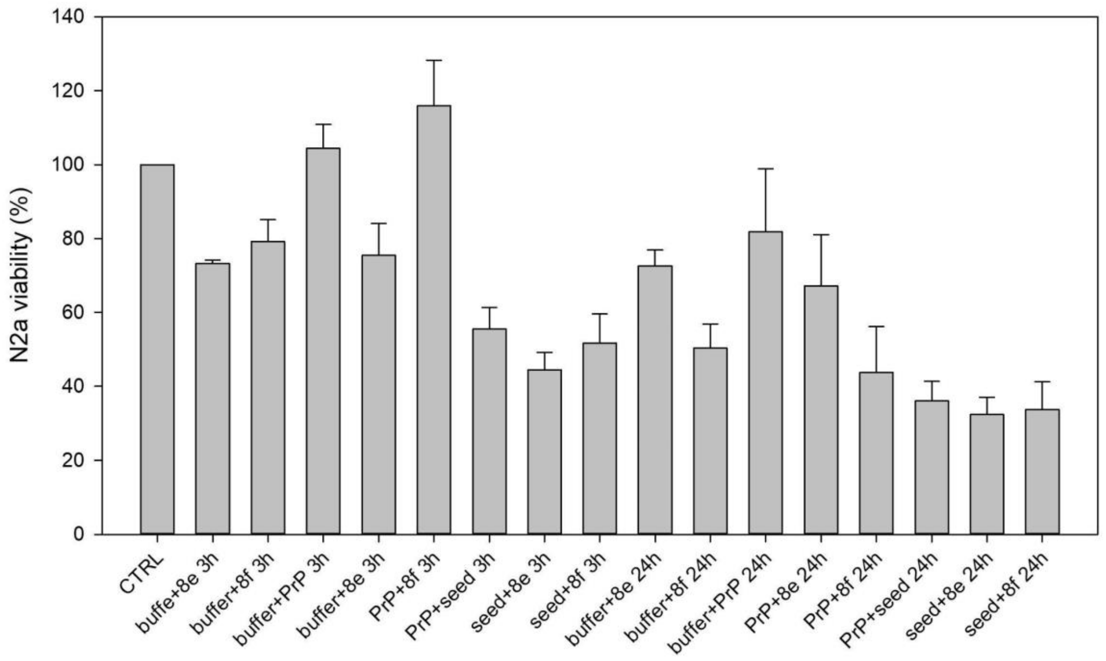

Figure S51. N2a cells cell viability in the presence of aggregates produced in RT-QuIC assays using recombinant PrP23-231 as substrate in the absence or presence of PrP23-231 fibril seed, treated with **8e** or **8f**. Data are represented as the mean of four independent experiments, and error bars represent the standard error.
